# Alternate day fasting aggravates atherosclerosis through the suppression of hepatic ATF3 in *Apoe^−/−^* mice

**DOI:** 10.1093/lifemeta/loae009

**Published:** 2024-03-07

**Authors:** Yajuan Deng, Xiaoyu Yang, Xueru Ye, Youwen Yuan, Yanan Zhang, Fei Teng, Danming You, Xuan Zhou, Wenhui Liu, Kangli Li, Shenjian Luo, Zhi Yang, Ruxin Chen, Guojun Shi, Jin Li, Huijie Zhang

**Affiliations:** Department of Endocrinology and Metabolism, Nanfang Hospital, Southern Medical University, Guangzhou, Guangdong 510515, China; Department of Endocrinology and Metabolism, Nanfang Hospital, Southern Medical University, Guangzhou, Guangdong 510515, China; Department of Endocrinology and Metabolism, Nanfang Hospital, Southern Medical University, Guangzhou, Guangdong 510515, China; Department of Endocrinology and Metabolism, Nanfang Hospital, Southern Medical University, Guangzhou, Guangdong 510515, China; Department of Endocrinology and Metabolism, Nanfang Hospital, Southern Medical University, Guangzhou, Guangdong 510515, China; Department of Endocrinology and Metabolism, Nanfang Hospital, Southern Medical University, Guangzhou, Guangdong 510515, China; Department of Endocrinology and Metabolism, Nanfang Hospital, Southern Medical University, Guangzhou, Guangdong 510515, China; Department of Endocrinology and Metabolism, Nanfang Hospital, Southern Medical University, Guangzhou, Guangdong 510515, China; Department of Endocrinology and Metabolism, Nanfang Hospital, Southern Medical University, Guangzhou, Guangdong 510515, China; Department of Endocrinology and Metabolism, Nanfang Hospital, Southern Medical University, Guangzhou, Guangdong 510515, China; Department of Endocrinology and Metabolism, Nanfang Hospital, Southern Medical University, Guangzhou, Guangdong 510515, China; Department of Endocrinology and Metabolism, Nanfang Hospital, Southern Medical University, Guangzhou, Guangdong 510515, China; Department of Endocrinology and Metabolism, Nanfang Hospital, Southern Medical University, Guangzhou, Guangdong 510515, China; Department of Endocrinology and Metabolism, Guangdong Provincial Key Laboratory of Diabetology, The Third Affiliated Hospital of Sun Yat-sen University, Guangzhou, Guangdong 510630, China; Department of Endocrinology, The Second Hospital of Shanxi Medical University, Shanxi Medical University, Taiyuan, Shanxi 030001, China; Department of Endocrinology and Metabolism, Nanfang Hospital, Southern Medical University, Guangzhou, Guangdong 510515, China; Guangdong Provincial Key Laboratory of Shock and Microcirculation, Nanfang Hospital, Southern Medical University, Guangzhou, Guangdong 510515, China; State Key Laboratory of Organ Failure Research, National Clinical Research Center for Kidney Disease, Nanfang Hospital, Southern Medical University, Guangzhou, Guangdong 510515, China

**Keywords:** alternate day fasting, atherosclerosis, cholesterol, integrated stress response, activating transcription factor 3

## Abstract

Atherosclerosis is the major contributor to cardiovascular mortality worldwide. Alternate day fasting (ADF) has gained growing attention due to its metabolic benefits. However, the effects of ADF on atherosclerotic plaque formation remain inconsistent and controversial in atherosclerotic animal models. The present study was designed to investigate the effects of ADF on atherosclerosis in apolipoprotein E-deficient (*Apoe*^*−/−*^) mice. Eleven-week-old male *Apoe*^*−/−*^ mice fed with Western diet (WD) were randomly grouped into *ad libitum* (AL) group and ADF group, and ADF aggravated both the early and advanced atherosclerotic lesion formation, which might be due to the disturbed cholesterol profiles caused by ADF intervention. ADF significantly altered cholesterol metabolism pathways and down-regulated integrated stress response (ISR) in the liver. The hepatic expression of activating transcription factor 3 (ATF3) was suppressed in mice treated with ADF and hepatocyte-specific overexpression of *Aft3* attenuated the effects of ADF on atherosclerotic plaque formation in *Apoe*^−/−^ mice. Moreover, the expression of ATF3 could be regulated by Krüppel-like factor 6 (KLF6) and both the expressions of ATF3 and KLF6 were regulated by hepatic cellular ISR pathway. In conclusion, ADF aggravates atherosclerosis progression in *Apoe*^*−/−*^ mice fed on WD. ADF inhibits the hepatic ISR signaling pathway and decreases the expression of KLF6, subsequently inhibiting ATF3 expression. The suppressed ATF3 expression in the liver mediates the deteriorated effects of ADF on atherosclerosis in *Apoe*^*−/−*^ mice. The findings suggest the potentially harmful effects when ADF intervention is applied to the population at high risk of atherosclerosis.

## Introduction

Atherosclerosis is the major contributor to the majority of cardiovascular mortality worldwide [1], but the current therapeutics are still unsatisfactory. Hypercholesterolemia and chronic inflammation are the main hallmarks of the progression of atherosclerosis. Increased circulating low-density lipoprotein cholesterol (LDL-C) is considered as a critical step in the initiating process of artery atheroma [2]. Although atherosclerosis is defined as the abnormal deposition of lipids in the vascular walls, accumulating evidence demonstrates that inflammation is involved in all phases of atherosclerosis. In atherosclerotic lesions, the infiltrating monocytes differentiate into lipid-laden macrophage/foam cells [3] and the vascular smooth muscle cells (VSMCs) migrate from the media to the intima [4], subsequently contributing to the progression of arteriostenosis. Diet-induced metabolic abnormalities including obesity, hyperglycemia, dyslipidemia, insulin resistance (IR), and non-alcoholic fatty liver disease (NAFLD) are considered as atherogenic risk factors [5, 6]. The global high prevalence of metabolic diseases due to altered lifestyle indicates an urgent clinical need to identify potential therapeutics to reduce the morbidity and mortality associated with atherosclerosis.

Intermittent fasting (IF), a nutritional intervention in which *ad libitum* (AL) feeding is alternated with fasting periods, has remarkable effects on health, aging, and disease [7]. Alternate day fasting (ADF), the most popular and representative regimen of IF, has gained growing attention due to its metabolic benefits. ADF can significantly promote weight loss, improve glucose tolerance, insulin sensitivity, and dyslipidemia, and alleviate NAFLD progression in mice [8–13]. Similar metabolic benefits are also observed in obese patients receiving ADF intervention [14–16]. All of these improved metabolic parameters are closely associated with the development of atherosclerosis. Besides, ADF can reduce systemic inflammation markers such as intercellular cell adhesion molecule-1 (ICAM-1) and tumor necrosis factor-α (TNF-α) in healthy and overweight adults [17, 18]. Meanwhile, ADF intervention is able to suppress inflammatory responses in multiple pathogenic processes including liver injury and neuroinflammation in animal models [19, 20]. However, the roles of ADF in obesity-related cardiovascular diseases remain to be addressed. In humans, the cardioprotective benefits of ADF are speculated based on the observations that ADF decreases coronary artery disease risk indicators in both obese and healthy adults [15–17]. On the contrary, the effects of ADF on atherosclerotic plaque formation remain inconsistent and controversial in atherosclerotic animal models [21–23].

In the present study, we explored the effects of ADF on the progression of atherosclerosis in atherogenic mice lacking apolipoprotein E (*Apoe*^*−/−*^). Our results demonstrated that ADF aggravates Western diet (WD)-induced atherosclerotic lesion formation. Therefore, we further investigated how ADF affected the development of atherosclerosis.

## Results

### ADF aggravates the early and advanced atherosclerotic lesion formation in *Apoe*^^*−/−*^^ mice

**Figure 1:**
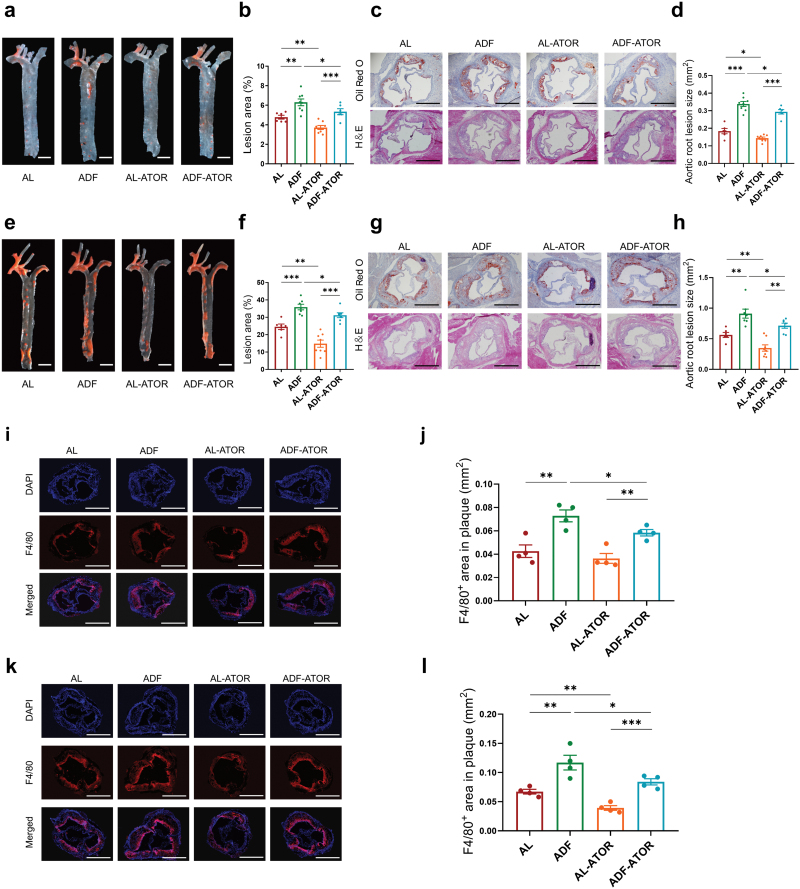
ADF aggravates the early and advanced atherosclerotic lesion formation in *Apoe*^*−/−*^ mice. Eleven-week-old male *Apoe*^*−/−*^ mice were fed with WD with or without 10 mg/kg body weight/d ATOR for 8 or 16 weeks. (a) Representative microscopic photographs of *en face* Oil Red O staining of aortas in *Apoe*^*−/−*^ mice fed with WD for 8 weeks. Scale bar (white), 500 μm. (b) Quantification of the *en face* atherosclerotic lesion areas of a (*n *= 6 to 8 mice per group). (c) Representative histological analysis of Oil Red O and H&E staining of aortic root sections in *Apoe*^*−/−*^ mice fed with WD for 8 weeks. Scale bar (black), 500 μm. (d) Quantification of the atherosclerotic lesion size of c (*n *= 6 to 8 mice per group). (e) Representative microscopic photographs of *en face* Oil Red O staining of aortas in *Apoe*^*−/−*^ mice fed with WD for 16 weeks. Scale bar (white), 500 μm. (f) Quantification of the *en face* atherosclerotic lesion areas of e (*n* = 6 to 8 mice per group). (g) Representative histological analysis of Oil Red O and H&E staining of aortic root sections in *Apoe*^*−/−*^ mice fed with WD for 16 weeks. Scale bar (black), 500 μm. (h) Quantification of the atherosclerotic lesion size of g (*n* = 6–8 mice per group). (i) Representative images of immunoﬂuorescent staining for F4/80 in aortic root cross sections in *Apoe*^*−/−*^ mice fed with WD for 8 weeks. Scale bar (white), 500 μm. (j) The calculated F4/80-positive areas in the plaques of i (*n* = 4 mice per group). (k) Representative images of immunoﬂuorescent staining for F4/80 in aortic root cross sections in *Apoe*^*−/−*^ mice fed with WD for 16 weeks. Scale bar (white), 500 μm. (l) The calculated F4/80-positive areas in the plaques of k (*n* = 4 mice per group). Data are presented as mean ± SEM. *P* values were determined by one-way ANOVA. ^*^P < 0.05, ^**^P < 0.01, ^***^P < 0.001.

To investigate the possible impact of ADF on the development of atherosclerosis, eleven-week-old male *Apoe*^*−/−*^ mice fed with WD were randomly grouped to *ad libitum* (AL) group and ADF group in the absence and presence of atorvastatin (ATOR). Mice in the AL group were allowed unrestricted access to food, while mice in the ADF group were fed with alternate 24-h feeding and 24-h fasting (Supplementary Fig. S1a). As demonstrated by Oil Red O staining of longitudinally opened aortas and hematoxylin and eosin (H&E) staining of aortic root regions, 8 weeks of ADF resulted in a 1.3-fold increase in lesion area and a 1.5-fold increase in lesion size compared with AL intervention (Fig. 1a–d). Additionally, *Apoe*^*−/−*^ mice were fed with WD for 16 weeks to establish an advanced atherosclerotic model characterized by expanded lesion size with necrotic cores. Consistently, the ADF group exhibited larger lesions in both the *en face* aortas (1.5-fold increase in lesion area) and sections of the aortic roots (1.4-fold increase in lesion size) (Fig. 1e–h). Similar trends of ADF on atherogenesis were also observed under ATOR intervention (Fig. 1a–h).

Besides, ADF significantly augmented the amounts of macrophages in atherosclerotic lesions both in the absence and presence of ATOR, as shown by the immunofluorescent staining of the macrophage marker F4/80 in both the early and advanced atherosclerosis (Fig. 1i–l). Meanwhile, increased accumulations of vascular muscle cells in aortic roots were also observed after ADF intervention both in the absence and presence of ATOR, as shown by the immunofluorescent staining of α-smooth muscle actin (α-SMA) in advanced atherosclerosis (Supplementary Fig. S1b–e). Taken together, these results demonstrated that the ADF regimen contributes to the aggravation of atherosclerotic progression in *Apoe*^*−/−*^ mice.

### ADF ameliorates WD-induced metabolic abnormalities in *Apoe*^*−/−*^ mice

**Figure 2:**
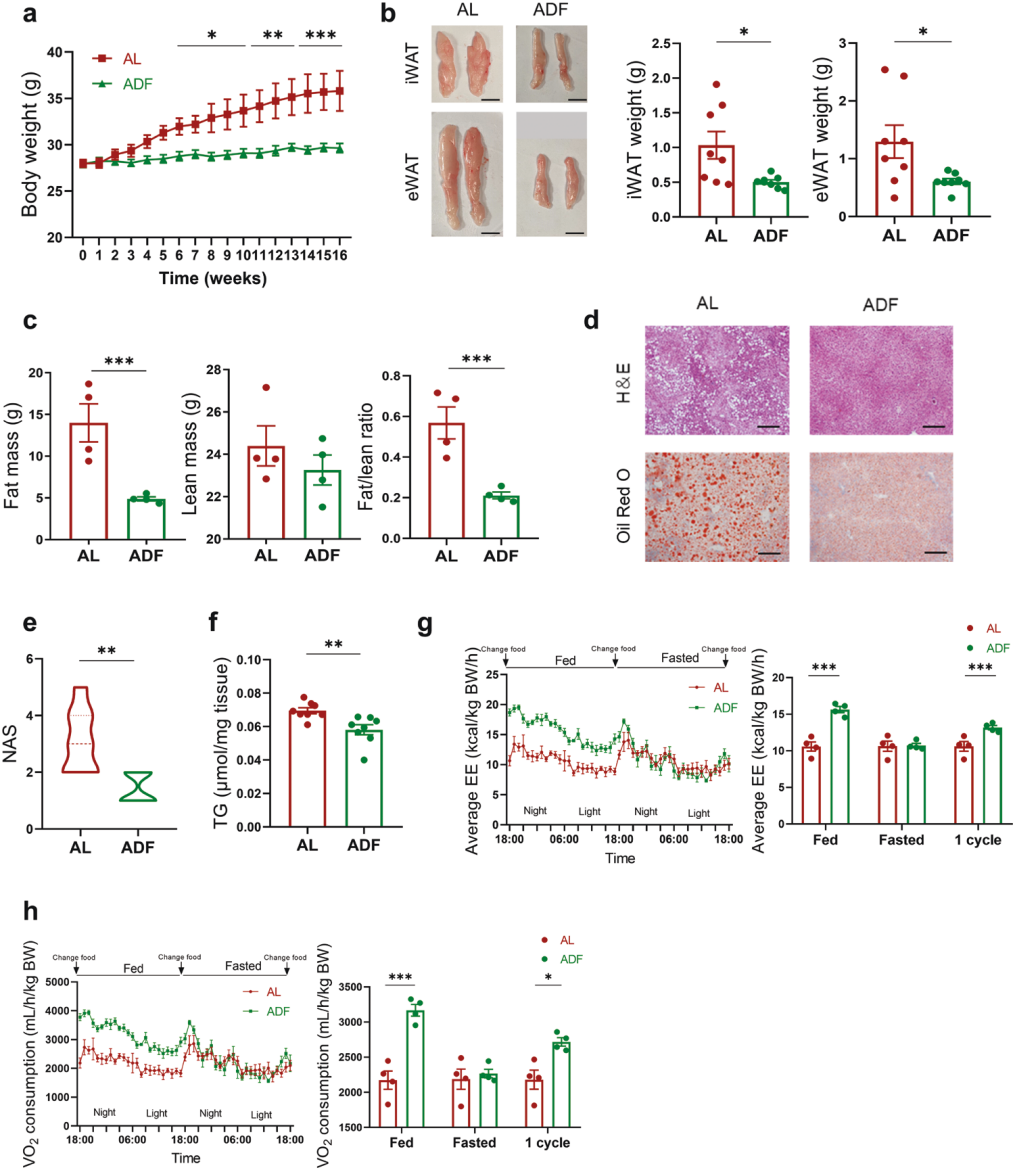
ADF ameliorates WD-induced metabolic abnormalities in *Apoe*^*−/−*^ mice. Eleven-week-old male *Apoe*^*−/−*^ mice fed on WD were treated with either AL or ADF intervention for 16 weeks. (a) Body weight measurement during 16 weeks of ADF (*n* = 6–8 mice per group). (b) Representative images and weight of iWAT and eWAT of the AL and ADF mice (*n* = 6–8 mice per group). Scale bar (black), 1 cm. (c) Body composition showing fat and lean mass (*n* = 4 mice per group). (d) H&E and Oil Red O staining of liver sections. Scale bar (black), 100 μm. (e) NAS (*n* = 6–8 mice per group). (f) Liver TG contents (*n* = 6–8 mice per group). (g and h) Average EE (g) and O_2_ consumption (h) during one cycle of ADF were expressed relative to unit body weight (*n* = 4 mice per group). On fed days, both groups had *ad libitum* access to food. In fasted days, the ADF mice were fasted while the AL mice had free access to food. ‘‘1 cycle’’ data present the average value of the fed and fasted days. Data are presented as mean ± SEM. *P* values were determined by a two-tailed unpaired Student’s *t*-test. ^*^P < 0.05, ^**^P < 0.01, ^***^P < 0.001.

Next, we explored the effect of a 16-week ADF regimen on WD-induced metabolic dysfunctions in *Apoe*^*−/−*^ mice. The ADF mice displayed pronounced lower body weight (Fig. 2a) and decreased food intake (Supplementary Fig. S2a) compared with the AL mice. ADF led to reduced amount of epididymal white adipose tissue (eWAT) and subcutaneous inguinal white adipose tissue (iWAT) (Fig. 2b). Meanwhile, body composition analysis further indicated that ADF significantly reduced fat mass without affecting lean mass (Fig. 2c). Histological examination of iWAT and eWAT revealed that ADF decreased adipocytes size (Supplementary Fig. S2b). Furthermore, ADF improved WD-induced glucose intolerance without affecting insulin sensitivity (Supplementary Fig. S2c and d) and ameliorated diet-induced fatty liver characterized by reduced intrahepatic lipid droplets, NAFLD activity score (NAS), total triglyceride (TG) contents, serum alanine transaminase (ALT) and aspartate aminotransferase (AST) levels, and inflammatory cytokine levels (Fig. 2d–f; Supplementary Fig. S2e and f). Chronic inflammation is the main hallmark in the development of atherosclerosis, and circulating proinflammatory cytokines were measured. However, there were no significant differences in TNF-α, soluble TNF receptor 2 (sTNFR2), and interleukin-1β (IL-1β) between AL and ADF groups (Supplementary Fig. S2g).

To determine the adaptive metabolic changes in energy homeostasis induced by ADF, indirect calorimetry was applied. The average energy expenditure (EE), VO_2_ consumption, VCO_2_ exhalation, and locomotor activity in the ADF mice were higher than that of the AL group on the fed day and during the entire cycle (Fig. 2g and h; Supplementary Fig. S3a–c). Respiratory exchange ratios (RERs) of the ADF mice were significantly lower than that of their AL counterparts on the fasted day and during the whole ADF cycle (Supplementary Fig. S3d), indicating the increased utilization of lipids as energy fuel in ADF mice. Interestingly, gene expressions involved in thermogenesis, such as cell death-inducing DNA fragmentation factor-like effector A (*Cidea*) and peroxisome proliferator-activated receptor-γ coactivator-1α (*Pgc1*α) in eWAT, and uncoupling protein 1 (*Ucp1*) and *Cidea* in iWAT, were increased under ADF intervention in *Apoe*^*−/−*^ mice (Supplementary Fig. S3e and f). Collectively, the above results showed that ADF protects *Apoe*^*−/−*^ mice from WD-induced obesity, hyperglycemia, and fatty liver, and promotes energy expenditure.

### ADF deteriorates cholesterol profiles in *Apoe*^*−/−*^ mice

**Figure 3:**
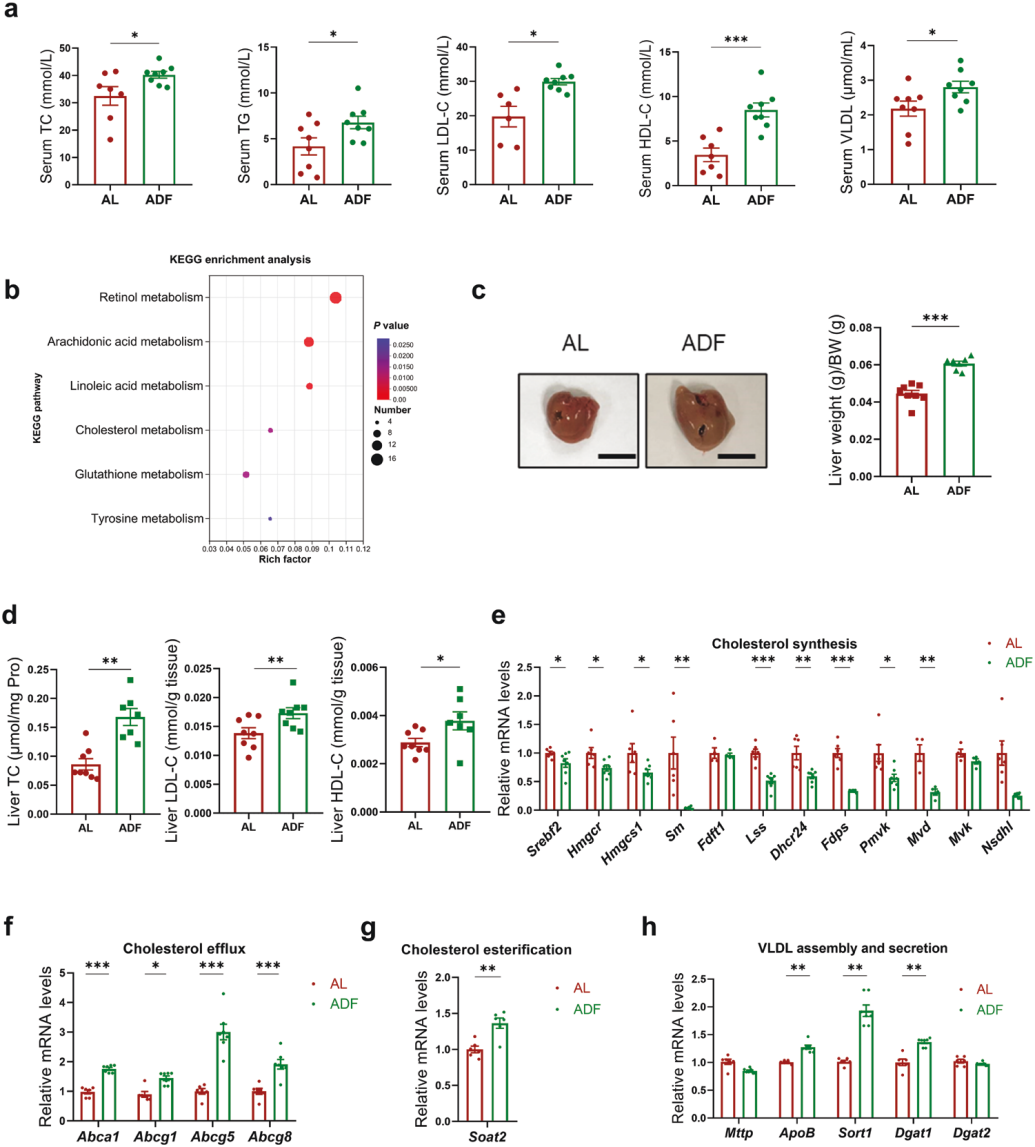
ADF deteriorates cholesterol profiles in *Apoe*^*−/−*^ mice. Eleven-week-old male *Apoe*^*−/−*^ mice fed on WD were treated with either AL or ADF intervention for 16 weeks. (a) Serum TC, TG, LDL-C, HDL-C, and VLDL levels from the AL and ADF mice (*n *= 6–8 mice per group). (b) Hepatic transcriptome analysis: KEGG pathway enrichment analysis of DEGs between the AL and ADF group. A default *P* value of < 0.05 was considered statistically significant with a fold-change > 1.5. (c) Representative photographs of livers and liver weight-to-body weight ratios of the AL and ADF mice (*n* = 6–8 mice per group). Scale bar (black), 1cm. (d) TC, LDL-C, and HDL-C levels in the livers of the AL and ADF mice (*n* = 6–8 mice per group). (e–h) The expressions of genes involved in cholesterol synthesis (e), cholesterol efflux (f), cholesterol esterification (g), and VLDL assembly/secretion (h) in the livers of the AL and ADF mice were evaluated by RT-qPCR (*n* = 6–8 mice per group). Data are presented as mean ± SEM. *P* values were determined by a two-tailed unpaired Student’s *t*-test. ^*^P < 0.05, ^**^P < 0.01, ^***^P < 0.001.

Lipid accumulation is a key hallmark during the progression of atherosclerosis, and lipid metabolism imbalance acts as the leading cause [24]. We found that ADF treatment deteriorated lipid profiles in *Apoe*^*−/−*^ mice, as circulating total cholesterol (TC), TG, LDL-C, high-density lipoprotein cholesterol (HDL-C), and very low-density lipoprotein (VLDL) levels were increased after a 16-week ADF intervention (Fig. 3a). The data indicated that the deteriorated cholesterol profiles might mediate the proatherogenic effects of ADF regimen in *Apoe*^*−/−*^ mice. Given that the liver plays a pivotal role in cholesterol metabolism and is one of the most affected organs during ADF, we performed transcriptome analysis by RNA sequencing (RNA-seq) on the livers from *Apoe*^*−/−*^ mice subjected to ADF or AL. ADF induced a global change in hepatic transcriptome and 786 genes were differentially expressed among the two groups. Kyoto Encyclopedia of Gene and Genomes (KEGG) pathway enrichment analysis of differentially expressed genes (DEGs) revealed that the significantly enriched metabolism pathways involved cholesterol metabolism (Fig. 3b). ADF intervention led to an increased liver weight and hepatic cholesterol content (Fig. 3c and d). Cholesterol metabolism is controlled by a feedback mechanism. Key genes involved in cholesterol biogenesis such as sterol regulatory element binding factor 2 (*Srebf2*), 3-hydroxy-3-methylglutaryl-CoA synthase 1 (*Hmgcs1*), 3-hydroxy-3-methylglutaryl-CoA reductase (*Hmgcr*), and squalene monooxygenase (*Sm*) were reduced under ADF intervention (Fig. 3e). Meanwhile, genes involved in cholesterol efflux, esterification, and VLDL assembly/secretion were increased (Fig. 3f–h).

Since the proximal jejunum is the major tissue for cholesterol absorption, we measured TC levels and the mRNA levels of representative genes involved in cholesterol absorption in proximal jejunum of the AL and ADF mice. No significant differences were observed in the expressions of Niemann-Pick C1-like 1 (*Npc1l1*), ATP-binding cassette transporter A1 (*Abca1*), scavenger receptor group B type 1 (*Srbi*), ATP-binding cassette transporter G8 (*Abcg8*), ATP-binding cassette transporter G5 (*Abcg5*), and sterol *O*-acyltransferase 2 (*Soat2*) in the ADF mice compared to that of the AL mice (Supplementary Fig. S4a). Consistently, cholesterol levels were comparable between the AL and ADF mice (Supplementary Fig. S4b). These data suggest that ADF may not affect intestinal cholesterol absorption.

To exclude the impact of sex hormones on atherosclerosis and lipid profiles, we conducted additional animal experiments using female *Apoe*^*−/−*^ mice. Eight weeks of ADF regimen also significantly lowered body weight (Supplementary Fig. S5a). Consistently, ADF intervention led to a significant increase in atherosclerotic lesion area (Supplementary Fig. S5b and c) and lesion size of aortic roots (Supplementary Fig. S5d and e) compared with the AL group. Meanwhile, ADF intervention led to an increased liver weight (Supplementary Fig. S5f) and hepatic cholesterol content (Supplementary Fig. S5g), as well as elevated serum TC, TG, LDL-C, and HDL-C levels (Supplementary Fig. S5h). The above results indicated that 8 weeks of ADF regimen also exhibited similar aggravated effects on cholesterol profiles and atherosclerosis in female *Apoe*^*−/−*^ mice. We postulated that ADF deteriorates cholesterol profiles and thus promotes the progression of atherosclerosis in both male and female *Apoe*^*−/−*^ mice.

### ADF decreases hepatic ATF3 expression in *Apoe*^*−/−*^ mice

**Figure 4:**
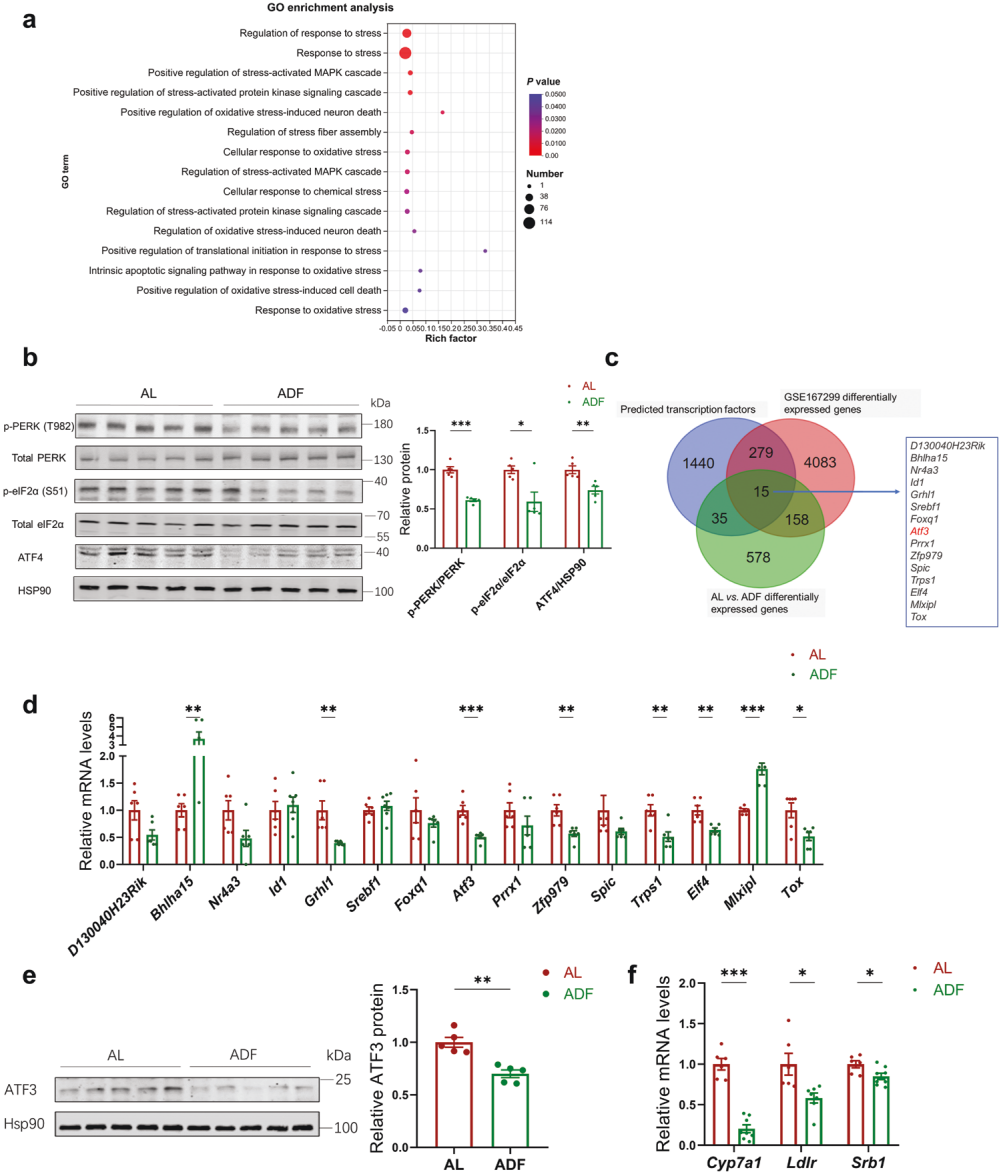
ADF decreases hepatic ATF3 expression in *Apoe*^*−/−*^ mice. (a) Hepatic transcriptome analysis of enriched stress-related GO terms for down-regulated genes in the livers of the ADF mice. (b) Western blot analysis of hepatic ISR components in the AL and ADF mice fed on WD for 16 weeks (*n* = 5 mice per group). (c) Venn diagram showing the overlap of three screened lists. GSE167299: microarray of liver from Tn-injected C57BL/6 mice. (d) Relative mRNA levels of candidate genes in the liver from the AL and ADF mice evaluated by RT-qPCR (*n* = 6–8 mice per group). (e) Relative protein levels of ATF3 in the livers of the AL and ADF mice fed with WD for 16 weeks were evaluated by western blot analysis (*n* = 5 mice per group). (f) Relative mRNA levels of ATF3 target genes in the livers of AL and ADF mice fed on WD for 16 weeks were evaluated by RT-qPCR (*n* = 6–8 mice per group). Data are presented as mean ± SEM. *P* values were determined by a two-tailed unpaired Student’s *t*-test. ^*^P < 0.05, ^**^P < 0.01, ^***^P < 0.001.

Next, we aimed to further explore the potential regulator in hepatic cholesterol metabolism during ADF intervention. We analyzed the DEGs between the AL and ADF groups by using gene ontology (GO) enrichment analysis and we observed that ADF down-regulated stress-related gene sets (Fig. 4a). The integrated stress response (ISR) is an elaborate signaling pathway, which is activated in response to a range of cell intrinsic and extrinsic stresses [25]. The core event in ISR is the phosphorylation of eukaryotic translation initiation factor 2α (eIF2α) on serine 51, which leads to the induction of the transcription factor activating transcription factor 4 (ATF4) [26]. We further investigated whether ADF could affect hepatic ISR. Western blotting analysis revealed that phosphorylation of protein kinase R (PKR)-like endoplasmic reticulum kinase (PERK) and eIF2α was markedly decreased in the livers of the ADF mice, along with the suppression of ATF4 expression, suggesting that ADF intervention inhibited the hepatic ISR signaling pathway (Fig. 4b). Previous study revealed that hepatic metabolic response caused by IF involves changes in multiple genes and pathways, among which transcription factors may play a key role. Further, we screened the candidates of hepatic transcription factors which satisfied all of the following criteria: (i) The gene is differentially expressed between the AL and ADF groups; (ii) The gene is predicted as a transcription factor according to AnimalTFDB database; (iii) The gene expression is regulated by hepatic stress (NCBI GEO: GSE167299). The DEGs from the three categories were merged and finally 15 overlapped candidates were screened (Fig. 4c). The expressions of these genes in our samples were further validated using reverse transcription quantitative real-time polymerase chain reaction (RT-qPCR) (Fig. 4d). Among them, the expression of activating transcription factor 3 (*Atf3*) was the most significantly decreased one in the liver from the ADF mice, which was further verified by western blotting (Fig. 4d and e). Given that hepatic ATF3 has been identified to modulate lipoprotein metabolism by inducing the expressions of cholesterol 7α-hydroxylase (CYP7A1), LDL receptor (LDLR), and scavenger receptor group B type 1 (SR-B1) [27], we further observed that the mRNA levels of *Cyp7a1*, *Ldlr,* and *Srb1* were reduced in the liver of the ADF *Apoe*^*−/−*^ mice (Fig. 4f). Hence, these results suggested that ADF leads to a reduction of hepatic ATF3 expression in *Apoe*^*−/−*^ mice.

### Hepatocyte-specific overexpression of *Atf3* attenuates the effects of ADF on atherosclerotic plaque formation

**Figure 5:**
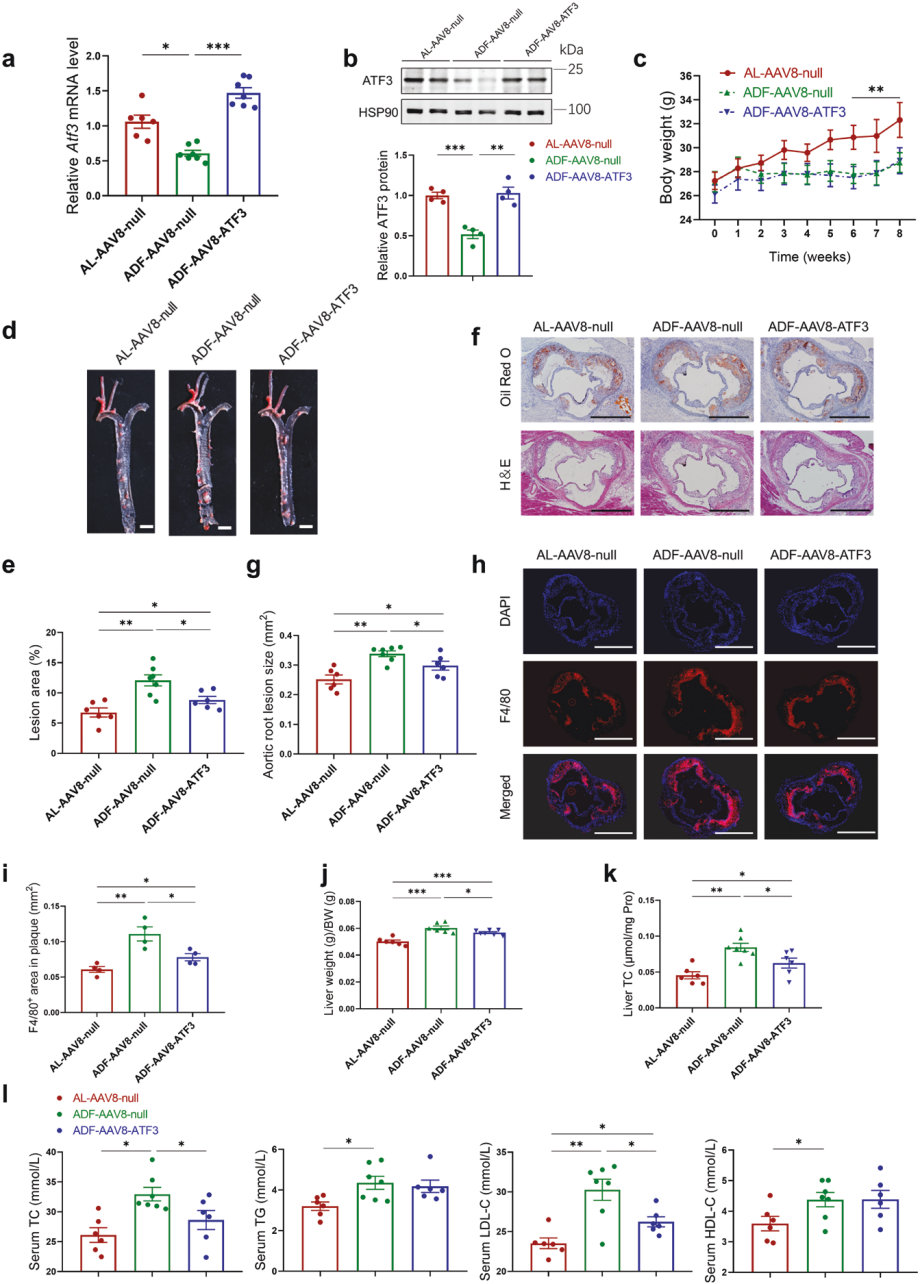
Hepatocyte-specific overexpression of *Atf3* attenuates the effects of ADF on atherosclerotic plaque formation. Eleven-week-old male *Apoe*^*−/−*^ mice were intravenously injected with either AAV8-TBG-null or AAV8-TBG-ATF3 and were then fed on WD with AL or ADF regimen 1 week after injection. After 8 weeks of ADF regimen, mice were sacrificed. (a) Relative hepatic *Atf3* mRNA levels determined by RT-qPCR. (b) Relative hepatic ATF3 protein levels determined by western blot analysis. (c) Body weight measurement during 8 weeks of ADF regimen (*n* = 6–7 mice per group). (d) Representative microscopic photographs of *en face* Oil Red O staining of aortas. Scale bar (white), 500 μm. (e) Quantification of the *en face* atherosclerotic lesion areas of d (*n* = 6 to 7 mice per group). (f) Representative histological analysis of Oil Red O and H&E staining of aortic root cross sections. Scale bar (black), 500 μm. (g) Quantification of lesion sizes in aortic root sections of f (*n* = 6–7 mice per group). (h) Representative images of immunofluorescent staining for F4/80 in aortic root cross sections. Scale bar (white), 500 μm. (i) Calculated F4/80-positive areas in the plaques of h (*n *= 4 mice per group). (j and k) Liver weight-to-body weight ratios (j) and total hepatic cholesterol levels (k) of the AL and ADF mice (*n* = 6–7 mice per group). (l) Serum TC, TG, LDL-C, and HDL-C levels from indicated groups (*n* = 6–7 mice per group). Data are presented as mean ± SEM. *P* values were determined by one-way ANOVA. ^*^P < 0.05, ^**^P < 0.01, ^***^P < 0.001.

To verify whether hepatic ATF3 primarily contributed to the progression of atherosclerosis in *Apoe*^*−/−*^ mice under ADF intervention, an adeno-associated virus (AAV) for expression of mouse *Atf3* under the control of a thyroxine binding globulin (TBG) promoter (AAV8-TBG-ATF3) was intravenously (i.v.) injected into ADF *Apoe*^*−/−*^ mice fed with WD. AAV8-based infection restored ATF3 expression in the liver during the ADF process (Fig. 5a and b), without affecting food intake or body weight (Fig. 5c; Supplementary Fig. S6a). The TBG promoter resulted in liver-specific delivery of ATF3 (Supplementary Fig. S6b). Consistent with previous findings, the ADF mice infected with the control vector showed reduced body weight but worsened atherosclerosis, local macrophage accumulation, and liver and serum cholesterol profiles compared to the AL mice infected with the control vector (Fig. 5d−l). Compared to the AAV8-null group with ADF intervention, overexpression of *Atf3* in hepatocytes of ADF *Apoe*^*−/−*^ mice reduced the plaque area of *en face* aortas by 24% (Fig. 5d and e), the plaque size of aortic roots by 12% (Fig. 5f and g), and the macrophage infiltration by 30% (Fig. 5h and i) in the lesions of aortic roots. Correspondingly, ADF-AAV8-ATF3 mice had decreased liver weight (Fig. 5j), hepatic cholesterol contents (Fig. 5k), and serum levels of TC and LDL-C (Fig. 5l) compared with ADF-AAV8-null mice. Meanwhile, the hepatic expressions of *Cyp7a1* and *Ldlr* were increased by *Atf3* overexpression (Supplementary Fig. S6c). All these data suggested that hepatocyte-specific overexpression of *Atf3* weakens the increased atherosclerotic lesion induced by ADF intervention.

### ADF inhibits the hepatic ISR signaling pathway and decreases the expressions of KLF6 and ATF3

**Figure 6:**
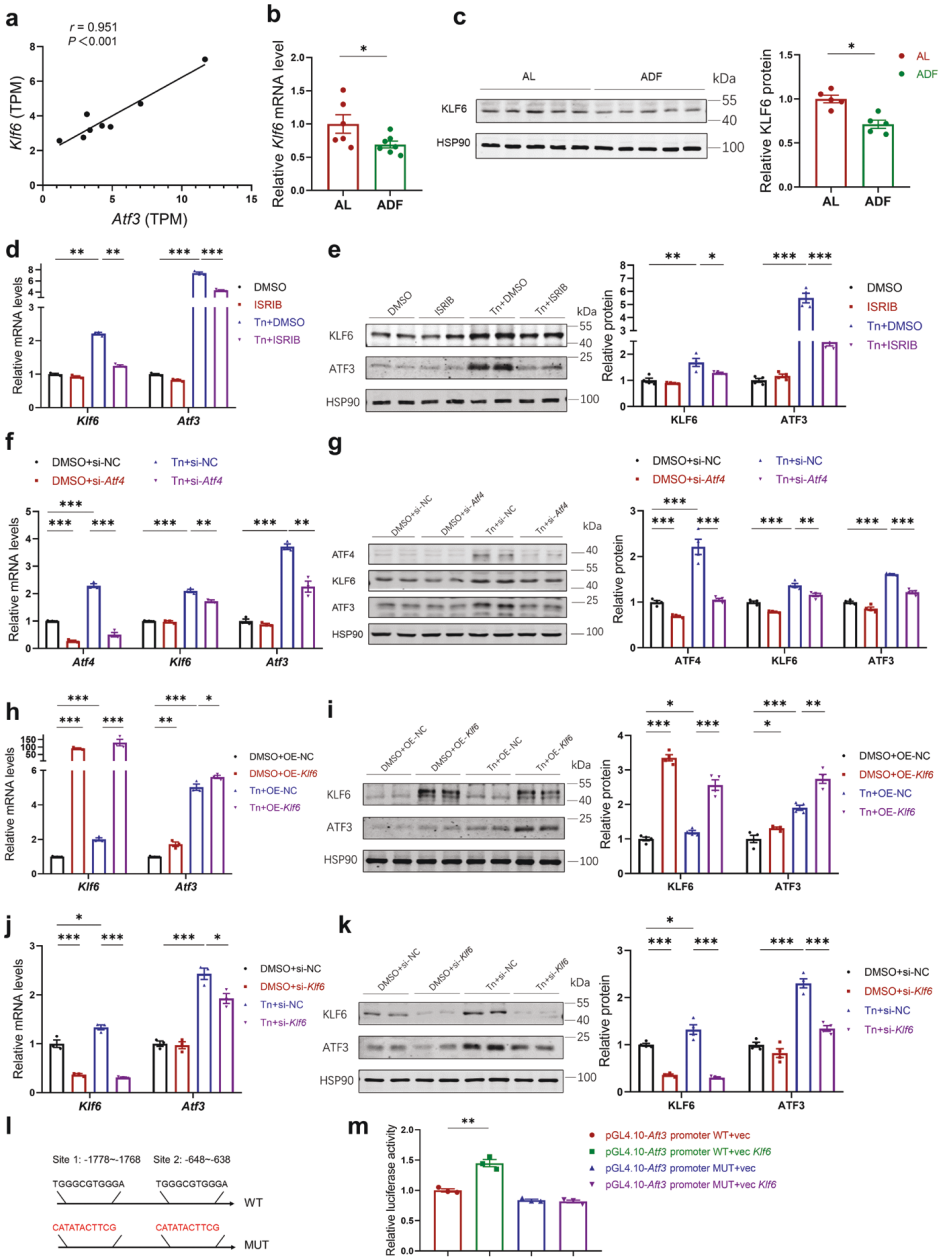
ADF inhibits the hepatic ISR signaling pathway and decreases the expressions of KLF6 and ATF3. (a) Association between the TPM expression levels of *Klf6* and *Atf3* in hepatic transcriptome analysis. (b and c) Relative *Klf6* mRNA levels (b) (*n* = 6–8 mice per group) and KLF6 protein levels (c) (*n* = 5 mice per group) in the livers of the AL and ADF mice fed on WD for 16 weeks. (d and e) Relative KLF6 and ATF3 mRNA (d) and protein levels (e) in Hepa1-6 cells pre-treated for 1 h with eIF2α inhibitor ISRIB (200 nmol/L) and then co-treated with Tn (5 mg/mL) for a further 6 h (*n* = 3 to 4 per group). (f and g) Relative ATF4, KLF6, and ATF3 mRNA (f) and protein levels (g) in Hepa1-6 cells transfected with control siRNA or *Atf4* siRNA and treated with Tn (5 mg/mL) for 6 h (*n* = 3–4 per group). (h and i) Relative KLF6 and ATF3 mRNA (h) and protein levels (i) in Hepa1-6 cells transfected with control vectors or *Klf6* overexpression plasmids and treated with Tn (5 mg/mL) for 6 h (*n* = 3–4 per group). (j and k) Relative KLF6 and ATF3 mRNA (j) and protein levels (k) in Hepa1-6 cells transfected with control siRNA and *Klf6* siRNA and treated with Tn (5 mg/mL) for 6 h (*n* = 3–4 per group). (l) The proximal promoter region of the mouse *Atf3* gene contains potential binding motifs for KLF6, and two of the binding sites were mutated and highlighted in red. (m) Luciferase reporter assays. Hepa1-6 cells were co-transfected with *Klf6* expression plasmids and luciferase reporter plasmids containing wild-type or mutant *Atf3* promoters for 48 h, together with a plasmid containing the *Renilla* luciferase gene to serve as a reference for transfection efficiency (*n* = 3 per group). Data are presented as mean ± SEM. Pearson correlation analysis was used (a). *P* values were determined by two-tailed unpaired Student’s *t*-test (b and c) and one-way ANOVA (d–k and m). ^*^P < 0.05, ^**^P < 0.01, ^***^P < 0.001.

The expression of ATF3 is triggered by numerous signals, including physiological stresses [28]. According to what we observed previously, ADF intervention suppressed the hepatic ISR signaling pathway, which raised our interest in exploring the novel mechanism of how ISR regulated ATF3 expression. In our RNA-seq data, we observed a significant and robust correlation between *Atf3* and Krüppel-like factor 6 (*Klf6*) expression (Fig. 6a). Besides, mRNA microarray analyses from previous studies indicated that *Klf6* expression was changed in response to several ISR inducers [29, 30]. KLF6 is a zinc finger transcription factor and a tumor suppressor, and was reported to directly bind to and activate the *Aft3* promoter in prostate cancer cells [31]. However, the regulative effects of KLF6 on ATF3 in hepatic ISR remain unknown.

We found that ADF intervention reduced KLF6 expression in the livers of *Apoe*^*−/−*^ mice (Fig. 6b and c), which was consistent with the alterations of ATF3 expression during ADF intervention. To further explore whether KLF6 and ATF3 expressions can be regulated by ISR, we detected changes in KLF6 and ATF3 expressions in mouse hepatocytes (Hepa1-6) treated with tunicamycin (Tn), an inhibitor of N-linked glycosylation as well as a well-characterized ISR stressor. Tn caused significant and potent induction of KLF6 and ATF3 expressions in Hepa1-6 cells (Fig. 6d and e). Meanwhile, ISR inhibitor (ISRIB), a drug-like small molecule that exerts its effects by inhibiting eIF2α phosphorylation in ISR [32] and acting as an ISR inhibitor, reversed both KLF6 and ATF3 expressions induced by Tn treatment (Fig. 6d and e). These data suggest that hepatic KLF6 and ATF3 expressions are regulated by the cellular ISR pathway, and ADF intervention inhibits the hepatic ISR signaling pathway and the expressions of KLF6 and ATF3. ATF4 is the core effector of ISR, and we found that knockdown of *Atf4* significantly attenuated the effects of Tn to induce the expressions of KLF6 and ATF3 (Fig. 6f and g). Therefore, we speculated that ADF inhibits the hepatic ISR and subsequently reduces the expression levels of KLF6 and ATF3 via ATF4.

Finally, to address the role of KLF6 in regulating ATF3 expression in hepatic ISR, overexpression or knockdown of *Klf6* in hepatocytes was performed. Vectors harboring *Klf6* expression significantly augmented both basal and Tn-induced ATF3 expression in Hepa1-6 cells (Fig. 6h and i). Furthermore, Tn-induced expression of ATF3 was abolished in Hepa1-6 cells transfected with small interfering RNA (siRNA)-mediated knockdown of *Klf6* (Fig. 6j and k). To further investigate whether KLF6 transcriptionally regulates *Atf3* expression in hepatocytes, we performed a reporter assay. Bioinformatic analysis obtained from the JASPAR database showed that the promoter region of the *Atf3* gene (nucleotide positions –2000 bp to +241 bp relative to the transcription start site) contains potential KLF6 binding sites. When this region was cloned and inserted into a luciferase reporter gene, transfection of a *Klf6* expression plasmid activated the luciferase activity in cultured Hepa1-6 cells (Fig. 6m). Mutation of the two potential binding sequences among the reporter construct clearly showed that *Klf6*-overexpression could not increase the transcriptional activity of the mutated *Atf3* promoter (Fig. 6l and m). These data demonstrated that KLF6 regulates ATF3 expression in hepatic ISR activation/inactivation.

## Discussion

ADF has been considered as a potentially superior alternative to traditional calorie restriction due to its metabolic benefits in recent years, while the effects of ADF on atherosclerosis have not been fully elucidated. The present study demonstrates that ADF aggravates WD-induced early and advanced atherosclerotic lesion formation in *Apoe*^−/−^ mice, which can be attributed to the disturbed cholesterol profiles caused by ADF intervention. Mechanistically, ADF inhibits the hepatic ISR signaling pathway and decreases the expression of KLF6, subsequently inhibiting ATF3 expression. The suppressed ATF3 expression in the liver mediates the deteriorated effects of ADF on atherosclerosis in *Apoe*^*−/−*^ mice.

Apolipoprotein E (ApoE) is required for efficient clearance of diet-derived chylomicrons and liver-derived VLDL remnants by the liver and adipose tissue [33, 34]. *Apoe*^−/−^ mice exhibit significant hypercholesterolemia and share a great similarity in the atherosclerotic plaque development to humans [35], establishing it as an excellent animal model for studying atherosclerosis. Previous studies regarding the cardioprotective benefits of IF are merely based on the improvement in risk indicators of cardiovascular diseases, while direct evidence showing the effects of IF on the development of atherosclerosis in animal models was paradoxical. Dorighello *et al*. reported that 12 weeks of ADF intervention induces obesity, diabetes, and hypercholesterolemia, thus worsening the development of atherosclerosis in LDLR knockout (*Ldlr*^−/−^) mice [21]. On the contrary, Chen *et al*. found that IF cycles consisting of 3-day feeding and 1-day fasting for 7 or 14 weeks substantially reduce atherosclerotic lesions in *Ldlr*^−/−^ mice [23]. What’s more, Inoue *et al*. showed that time-restricted feeding, another kind of IF, prevents diet-induced obesity but fails to ameliorate atherosclerosis in *Apoe*^−/−^ mice [22]. Those studies applied different mouse models, IF regimens, and diet components. In our experiments, we provided novel evidence that the ADF regimen improved atherogenic risk factors including obesity, hyperglycemia, and fatty liver, whereas deteriorated cholesterol profiles and accelerated the development of atherosclerosis in *Apoe*^−/−^ mice fed with WD. More importantly, such effects were sex-independent. Although ADF intervention did not affect intestinal cholesterol absorption, it did lead to decreased hepatic expressions of genes involved in cholesterol biogenesis. However, the levels of genes in cholesterol efflux, esterification, and VLDL assembly/secretion were increased, which may be mediated by a compensatory effect caused by cholesterol accumulation in the livers of ADF mice. In summary, the deterioration of cholesterol profiles under ADF intervention may contribute to worsened atherosclerotic lesion formation.

The 3 isoforms of human ApoE are encoded by 3 alleles, *ε2*, *ε3,* and *ε4*, respectively. Accumulating evidence suggests that the carriers of the *ε4* allele are associated with increased circulating LDL-C levels and a 42% higher risk of coronary heart disease [36, 37]. Nevertheless, prior clinical studies revealed that, instead of the decline in plasma lipid levels, plasma LDL-C levels increased by 13.4% (0.45 mmol/L) after 1 week-fasting in men who carried ApoE-*ε4* [38]. In healthy young adults, subjects with ApoE-*ε4*/*ε3* genotype had an increase of 0.31 ± 0.19 mmol/L in plasma LDL-C level after changing their diet from a monounsaturated fatty acid diet to a carbohydrate-rich diet, whereas subjects with ApoE-*ε3*/*ε3* and ApoE-*ε3*/*ε2* had unaltered and decreased LDL-C levels, respectively [39]. These results suggest the possibility that the function of ApoE may influence the effect of dietary intervention on plasma LDL-C. Given that the ADF regimen improved specific metabolic parameters but induced hypercholesterolemia in *Apoe*^−/−^ mice as we previously described, it must be cautious when ADF intervention is applied to the population carrying ApoE-*ε4* because of its potentially harmful effects on atherosclerosis. Further clinical studies are warranted to explore the effects of the ApoE genotype on response to ADF regimen. These findings have clinical implications for dietary intervention in the population at high risk.

ATF3 is a member of the ATF/cAMP response element binding protein (CREB) family and was demonstrated to have different functions in various metabolic organs, making it a master regulator in metabolic homeostasis [40]. ATF3 was reported to decrease the hepatic expression of gluconeogenic enzymes [41] and lipogenic genes while upregulating the expression of genes involved in lipolysis and browning in adipocytes [42]. ATF3 enhances hepatic HDL uptake and inhibits intestinal fat/cholesterol absorption [27]. Furthermore, hepatocyte-specific expression of ATF3 protects against hepatic cholesterol accumulation [43]. Based on those studies and our mRNA-seq analysis, we found that ADF could decrease the hepatic expression of ATF3 in *Apoe*^−/−^ mice. More importantly, such a phenomenon was observed due to a deterioration of cholesterol profiles caused by decreased hepatic ATF3 expression. Hepatocyte-specific overexpression of *Atf3* attenuated the effects of ADF on lipid metabolism. Meanwhile, the hepatic *Cyp7a1* and *Ldlr* expressions were increased by *Atf3* overexpression. Hepatic CYP7A1 is the rate-limiting enzyme in converting cholesterol into bile acids (BAs), and its up-regulation results in augmented BA excretion and corresponding reductions in TC and LDL-C [44]. LDLR regulates plasma LDL-C levels by mediating its internalization into the hepatocytes, and the increased LDLR levels could promote LDL-C clearance thus alleviating atherosclerosis [45]. The data suggest that hepatocyte-specific overexpression of *Atf3* exerts its protective effects in ADF mice by restoring the hepatic *Cyp7a1* and *Ldlr* expressions, indicating that ATF3 is one of the contributing factors that mediated the modulatory effects of ADF on hepatic cholesterol accumulation and circulating cholesterol profile.

ISR is one of the most important signaling pathways involved in cellular stresses [46]. The liver plays a key role in regulating cholesterol metabolism and responds to diet intervention. However, the physiological functions of hepatic ISR in metabolic diseases remain unclear [47]. In ISR pathways, PERK activation, as one of the canonical endoplasmic reticulum (ER) stress signaling pathways, selectively phosphorylates eIF2α and induces ATF4. ER stress-inducing agents were reported to cause sterol regulatory element binding protein (SREBP) activation and cholesterol accumulation [48, 49], while induction of the ISR by an artificial drug-activated eIF2α kinase reduces the level of SREBP and sterol-regulated mRNAs [50]. These studies suggest that how ISR components affect cholesterol homeostasis is still ambiguous. Our data indicated that ADF intervention inhibited the hepatic ISR signaling pathway, featuring the decreased phosphorylation of PERK and eIF2α and the reduced protein levels of ATF4. We also observed that hepatocellular ATF3 expression was regulated by the cellular ISR pathway, and hepatocyte-specific overexpression of *Atf3* in *Apoe*^−/−^ mice weakened the effects of ADF on cholesterol profiles and atherosclerosis. The above findings suggest that ATF3 could act as a downstream component of ISR in the liver, and its activation ameliorates hypercholesterolemia and atherosclerotic lesion formation.

In addition, a significant and robust correlation between *Atf3* and *Klf6* expression was observed based on our RNA-seq analysis. *Klf6*, an early-responsive gene to injury, serves as the responder of oxidative stress in pathological conditions [51, 52], and *Klf6* silencing attenuates oxidative stress-induced dysfunction [53]. Recently, the regulatory role of KLF6 in ATF3 expression was verified in endothelial cells [54], pancreatic cancer cells [55], and prostate cancer cells [31]. Our findings demonstrated that *Klf6* overexpression resulted in the induction of ATF3 in the absence and presence of ISR in Hepa1-6 cells. Correspondingly, KLF6 loss-of-function abolished ISR-induced ATF3 expression. Therefore, our results documented the changes in hepatic ATF3 expression and ISR pathway during ADF intervention *in vivo* and unraveled the previously unknown regulative effects of KLF6 on ATF3 in hepatic ISR *in vitro*.

Our research has several limitations. First, the deteriorated cholesterol profiles after ADF intervention were accompanied by the elevation of genes involved in cholesterol efflux, cholesterol esterification, and VLDL assembly/secretion in the liver. To validate the source of increased circulating cholesterol level, the rates of cholesterol synthesis in the liver should be measured using [^3^H] water. Meanwhile, the dual-isotope plasma ratio method should be applied to determine intestinal cholesterol absorption *in vivo*. Second, the regulative effects of KLF6 on ATF3 should be further validated in both ISR and ADF animal models. Third, due to the different lipid profile as compared to humans, the lipoprotein metabolism of *Apoe*^−/−^ mice could not completely resemble human pathophysiology. The obtained results cannot be directly extrapolated to humans, thus further clinical designs are needed to explore the effects of ADF regimen on atherosclerosis in real-world population studies.

To sum up, our study reveals that ADF could aggravate WD-induced atherosclerotic progression in *Apoe*^−/−^mice, and sheds light on the role of hepatic ATF3 in mediating the effects of ADF on atherosclerosis. Moreover, the study indicates that ADF inhibits the hepatic ISR signaling pathway, and provides evidence supporting the diverse effects of hepatic ISR in regulating cholesterol metabolism and atherosclerosis, raising interest in further exploration of ISR in metabolic disorders.

## Materials and methods

### Animals

*Apoe*^*−/−*^ mice on C57BL background were purchased from GemPharmatech Company. All mice were co-housed (four mice per cage) in a specific pathogen-free (SPF) facility in ventilated cages with controlled environment settings (22 ± 2 °C, 55 ± 15% humidity, 12-h light/12-h dark cycles). After 8 or 16 weeks of AL/ADF regimen, the mice were anesthetized by intraperitoneal injection of pentobarbital (Shanghai, China; 100 mg/kg body weight). The loss of reflex was detected by pricking the animal’s feet and legs with forceps, and then the mice were executed by cervical dislocation.

### ADF regimen and diets

*Apoe*^*−/−*^ mice aged 11 weeks were randomly divided into an AL group and an ADF group. These mice were fed with WD containing 42% fat and 0.2% cholesterol (Envigo, TD.88137) for 8 or 16 weeks. The AL mice were allowed unrestricted access to food, while the ADF mice were intervened with 1 day of feeding followed by 1 day of fasting. The food was removed at 6:00 p.m. and then provided again in the following day at 6:00 p.m. The clean bedding was replaced at the beginning of the fasting day to prevent foraging and coprophagia. Mice in the AL group were handled equivalently to control for any handling stress and minimize experimental variation between groups. All mice had free access to water. Daily food intake was measured by monitoring the weight of the remaining food. Body weight was measured weekly. Mice in the ADF group were sacrificed on the fasting day at the end of the experiments.

### Body composition and indirect calorimetry

Body fat and lean mass of live mice were assessed using mouse magnetic resonance imaging (MesoQMR23-060H-I, SUZHOU NIUMAG) following the manufacturer’s protocol. Whole body metabolic states were tested by indirect calorimetry on *Apoe*^*−/−*^ mice after 14-week of ADF treatment using a comprehensive lab animal monitoring system (CLAMS) (Promethion Metabolic Screening Systems, Sable Systems International, North LasVegas, NV, USA). Light and feeding conditions were kept the same as that in home cages. After 48-h acclimatization, mice were monitored for 24 h in the feeding state and then 24 h in the fasting state for recording data. Locomotor activity was determined at the same time using infrared beam interruption.

### Intraperitoneal glucose and insulin tolerance tests

Mice were fed according to AL or ADF regimens on WD. For intraperitoneal insulin tolerance test (ipITT), mice were injected intraperitoneally with insulin (0.75 IU/kg body weight) after a 4-h fasting. For intraperitoneal glucose tolerance test (ipGTT), mice were injected intraperitoneally with d-glucose (1.5 g/kg body weight) after a 16-h fasting. Blood glucose levels were measured at various time points using a glucometer (ACCUCHEK, Roche) by tail bleeds.

### AAVs

AAVs (AAV8) using TBG promoter for expression of mouse *Atf3* with C-terminal 3ʹ-Flag tags were constructed by Genechem Company. Each mouse was intravenously injected with AAV8-ATF3 or AAV8-null (control) (2 × 10^11^ plaque-forming units [pfu]) and then fed on WD with AL or ADF regimen 1 week after injection.

### Analysis of atherosclerotic plaques

Aortic arch, thoracic aorta, and left ventricular outflow tract samples were obtained after perfusion with phosphate-buffered saline (PBS) via the left ventricle. Samples were isolated and gently cleaned of the adventitia in PBS. *En face* aortas (aortic arch and thoracic aorta) were fixed in 4% formalin for 24 h and washed in PBS. Then the aortas were incubated in 60% isopropanol for 5 min and stained with Oil Red O for 60 min. After a short immersion in 60% isopropanol after the staining procedure, the aortas were washed in water for 1 min and photographed immediately. The left ventricular outflow tract samples were embedded in an optical coherence tomography (OCT) compound and frozen on dry ice to acquire frozen sections. Serial 10-μm-thick cryosections were collected from the middle portion of the ventricle to the aortic arch. The frozen sections were stained with H&E and Oil Red O after fixation with 4% formalin for 10 min. For immunofluorescent staining, the frozen sections were stained with antibodies against F4/80 (28463-1-AP, Proteintech) and α-SMA (BM0002, Boster), followed by incubation with Cy3-conjugated AffiniPure Goat Anti-Rabbit (111-165-003, Jackson) and Alexa Fluor® 488-conjugated AffiniPure Goat Anti-Rabbit (111-545-003, Jackson) as secondary antibodies. The quantification of lesion area or size was performed with ImageJ software.

### Serum and hepatic biochemical measurement

Blood was collected from the orbital venous plexus of mice. Liver lipids were extracted using TG and TC assay kits (APPLYGEN). Briefly, liver tissues (~20 mg) were weighed and homogenized, and then centrifuged at 3000 rpm for 5 min. Supernatants were collected and analyzed following the manufacturer’s instructions. Serum and hepatic TC and TG were measured using commercial kits (APPLYGEN). Serum TNF-α, sTNFR2, and IL-1β levels were measured using ELISA commercial kits from mlbio. Serum and hepatic LDL-C, HDL-C, VLDL, and serum ALT and AST levels were measured using commercial kits from Nanjing Jiancheng Bioengineering Institute.

### Histology analysis

Liver and WAT were harvested and fixed in 4% formalin for 24 h. Six-mm-thick paraffin-embedded sections were collected and stained with H&E. For Oil Red O staining, frozen sections were stained with Oil Red O (Solarbio) according to the standard procedures. Images were captured using a Leica microscope (Leica DMi8-M). NAS was calculated according to the Kleiner scoring system [56].

### Cell culture

Hepa1-6 cells were cultured in high glucose DMEM (Gibco) supplemented with 10% fetal bovine serum (FBS) (ExCell) and incubated at 37°C in a 5% CO_2_ atmosphere. Cells were evenly seeded onto well plates and incubated for 24 h when reaching 70% confluence. For *Klf6* overexpression, mouse *Klf6* cDNA was constructed in the pcDNA3.1 vector with 3ʹ-Flag tags. Hepa1-6 cells were transfected with the *Klf6* overexpression plasmids or control vector. To silence *Atf4* and *Klf6* expression in Hepa1-6 cells, a negative control siRNA and siRNA targeting *Atf4* (si-*Atf4*) and *Klf6* (si-*Klf6*) were synthesized by OBiO with the following sequences: si-NC 5ʹ-UUCUCCGAACGUG UCACGUTT-3ʹ, si-*Atf4* 5ʹ-CCAGAGCAUUCCUUUAGUUUATT-3ʹ, si-*Klf6* 5ʹ-CAGGAAAGUUUACACGAAATT-3ʹ. Transfection assays were performed with the Advanced DNA RNA Transfection Reagent (ZETA LIFE, AD600025) following the manufacturer’s instructions. Forty-eight hours after plasmid or siRNA transfection, cells were treated with 5 μg/mL Tn (MCE, HY-A0098) for 6 h and subsequently processed for RNA and protein expression analysis. To inhibit ISR, cells were pre-treated for 1 h with 200 nmol/L ISRIB (Beyotime, SC4332) [57] and then co-treated with Tn (5 μg/mL) for further 6 h.

### Luciferase reporter assay

The promoter region of the mouse *Atf3* gene extending from position −2000 bp (relative to the transcription start site) to +241 bp was cloned into the pGL4.10 vector (Promega) containing the firefly luciferase reporter gene. Mutant *Atf3* promotor was generated using the following primers: 5ʹ-GTGTGGAGCAGGGTG CATATACTTCGGACGGAGGGTGGGGCCCCGAGAGCCGTTTCC-3ʹ; 5ʹ-CGAGTTCGGGGGCCGCATATACTTCGGCACGAGGCTGCCG AATGTCTACG-3ʹ. For luciferase assay, Hepa1-6 cells were placed in 96-well plates and cotransfected with *Klf6* expression plasmids and luciferase reporter plasmids using Advanced DNA RNA Transfection Reagent (ZETA LIFE, AD600025) following the manufacturer’s instructions, together with a plasmid containing the *Renilla* luciferase gene to serve as a reference for transfection efficiency. After 48 h transfection, the firefly luciferase activity and *Renilla* luciferase activity were measured using the Dual Luciferase Reporter Gene Assay Kit (YEASEN, 11402ES60).

### RT-qPCR

Total RNA was extracted from cells or tissues using TRIzol (Accurate Biology). RNA concentration was determined using a Nanodrop analyzer (Thermo Scientific). Total RNA was transcribed into cDNA by a Reverse Transcription Kit (Accurate Biology) according to the manufacturer’s protocol. The amplification reactions were performed on a Light Cycler 480 (Roche) using a SYBR Real-time PCR Master Mix Kit (Yeason). Relative quantification analysis of gene expression was performed based on the 2^−ΔΔCt^ method and normalized to the expression of housekeeping gene 36B4 or 18S. Primers used for the analyses are shown in Supplementary Table S1.

### Western blot analysis

Total protein was extracted from tissues and cells in RIPA buffer (Beyotime) on ice. Protein concentrations were quantified by the BCA Kit (Thermo Fisher Scientific). The equivalent amount of protein was separated using SDS–PAGE and transferred to nitrocellulose membranes (Millipore). After blocking with 5% milk for 1 h at room temperature, the membranes were incubated with the indicated primary antibodies overnight at 4°C. On the second day, the membranes were incubated with secondary antibodies for 1 h at room temperature in the dark. Finally, the protein expression signals were visualized by Odyssey imaging systems (LI-COR). Antibodies used are listed in Supplementary Table S2.

### RNA-seq

High-throughput mRNA-seq was performed by Shanghai Majorbio. The RNA quality was determined by 2100 Bioanalyser (Agilent). RNA-seq transcriptome library was prepared following the TruSeq^TM^ RNA Sample Preparation Kit (Illumina). The libraries were sequenced with the Illumina HiSeq xten/NovaSeq 6000 sequencer (2 × 150 bp read length). The expression of each transcript was calculated according to the transcripts per million reads (TPM) method and differential expression analysis was performed using the DESeq2. Genes that were significantly changed by > 1.5-fold with a *P* value < 0.05 were considered to be significantly DEGs. GO and KEGG analyses of DEGs were performed between two groups of mice.

### Statistical analysis

All data were analyzed using SPSS 22.0 (IBM Corp). Values were shown as the mean ± standard error of the mean (SEM) or median (min–max). A two-tailed unpaired Student’s *t*-test was performed to test the differences between two groups. When more than two groups were compared, one-way analysis of variance (ANOVA) followed by Dunnett’s multiple comparisons was applied. Correlation analysis was performed using the Pearson correlation coefficient. Two-sided values of *P* < 0.05 were considered statistically significant. ^*^, ^**^, and ^***^ represent *P* < 0.05, *P* < 0.01, and *P* < 0.001, respectively.

## Supplementary Material

loae009_suppl_Supplementary_Material

## Data Availability

All study data are included in the article and/or Supplementary Materials. Materials are available upon request.
