# Supplementary material for: Alternate day fasting aggravates atherosclerosis through the suppression of hepatic ATF3 in Apoe−/− mice
Source: Life Metab. 2024 Mar 7;3(3):loae009. doi: 10.1093/lifemeta/loae009 (PMC11749235; doi:10.1093/lifemeta/loae009)
Supplement: loae009_suppl_Supplementary_Material [file loae009_suppl_Supplementary_Material.docx]

**Supplementary Material**

**Supplementary Figures S1-S6**


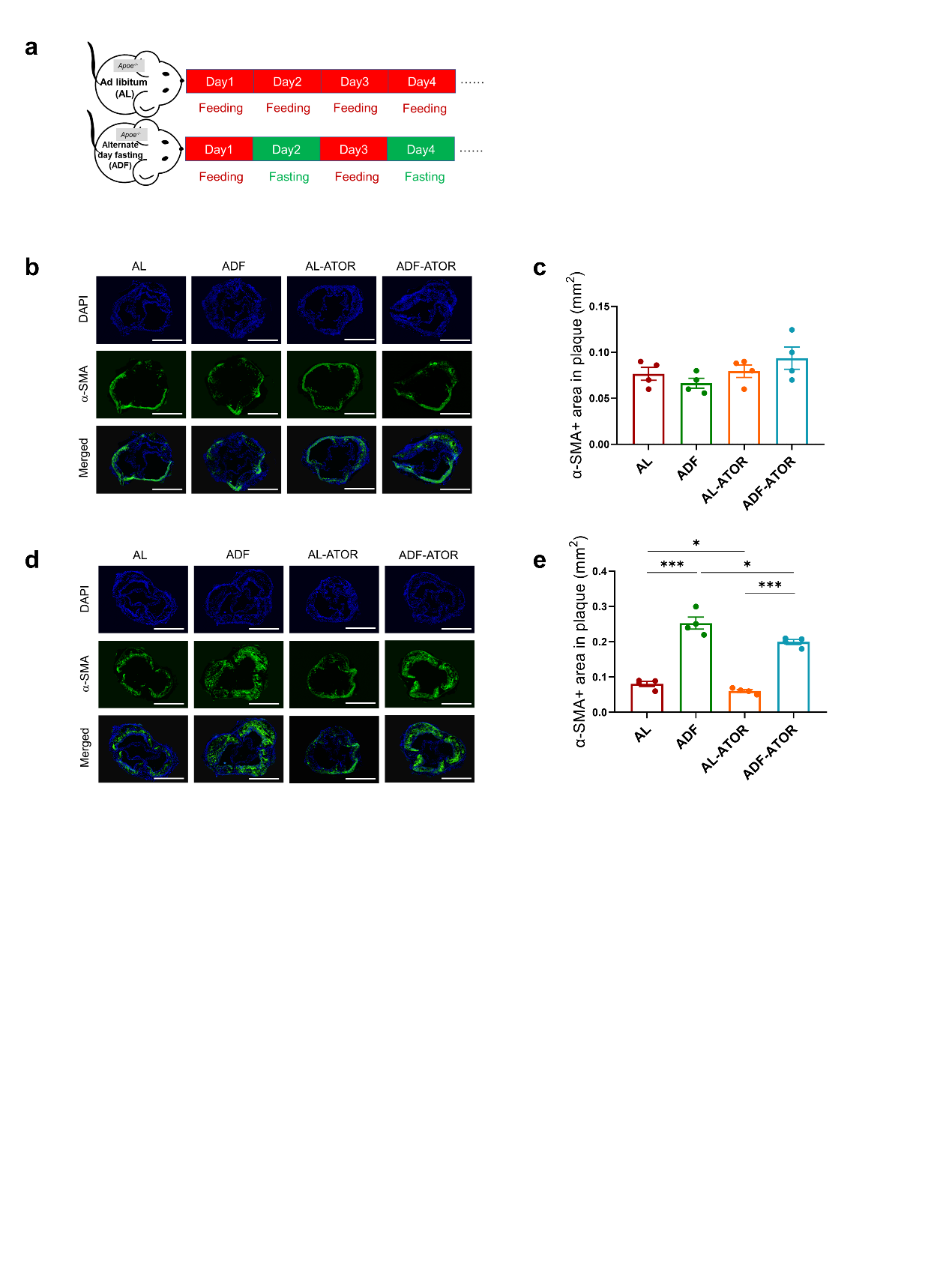


**Supplementary Figure S1** ADF increases the expression of α-SMA in atherosclerotic plaque of *Apoe^-/-^* mice. Eleven-week-old male *Apoe^-/-^* mice were fed with WD with or without 10mg/kg body weight/d ATOR for 8 or 16 weeks. (a) Schematic illustration of the ADF regimen. (b) Representative images of immunoﬂuorescent staining for α-SMA in aortic root cross sections in *Apoe^-/-^* mice fed with WD for 8 weeks. Scale bar (white), 500μm. (c) The calculated α-SMA-positive areas in the plaques of (b) (n=4 mice per group). (d) Representative images of immunoﬂuorescent staining for α-SMA in aortic root cross sections in *Apoe^-/-^* mice fed with WD for 16 weeks. Scale bar (white), 500μm. (e) The calculated α-SMA-positive areas in the plaques of (d) (n=4 mice per group). Data are presented as mean±SEM. *P* values are determined by one-way ANOVA. **P* < 0.05; *** *P*< 0.001.


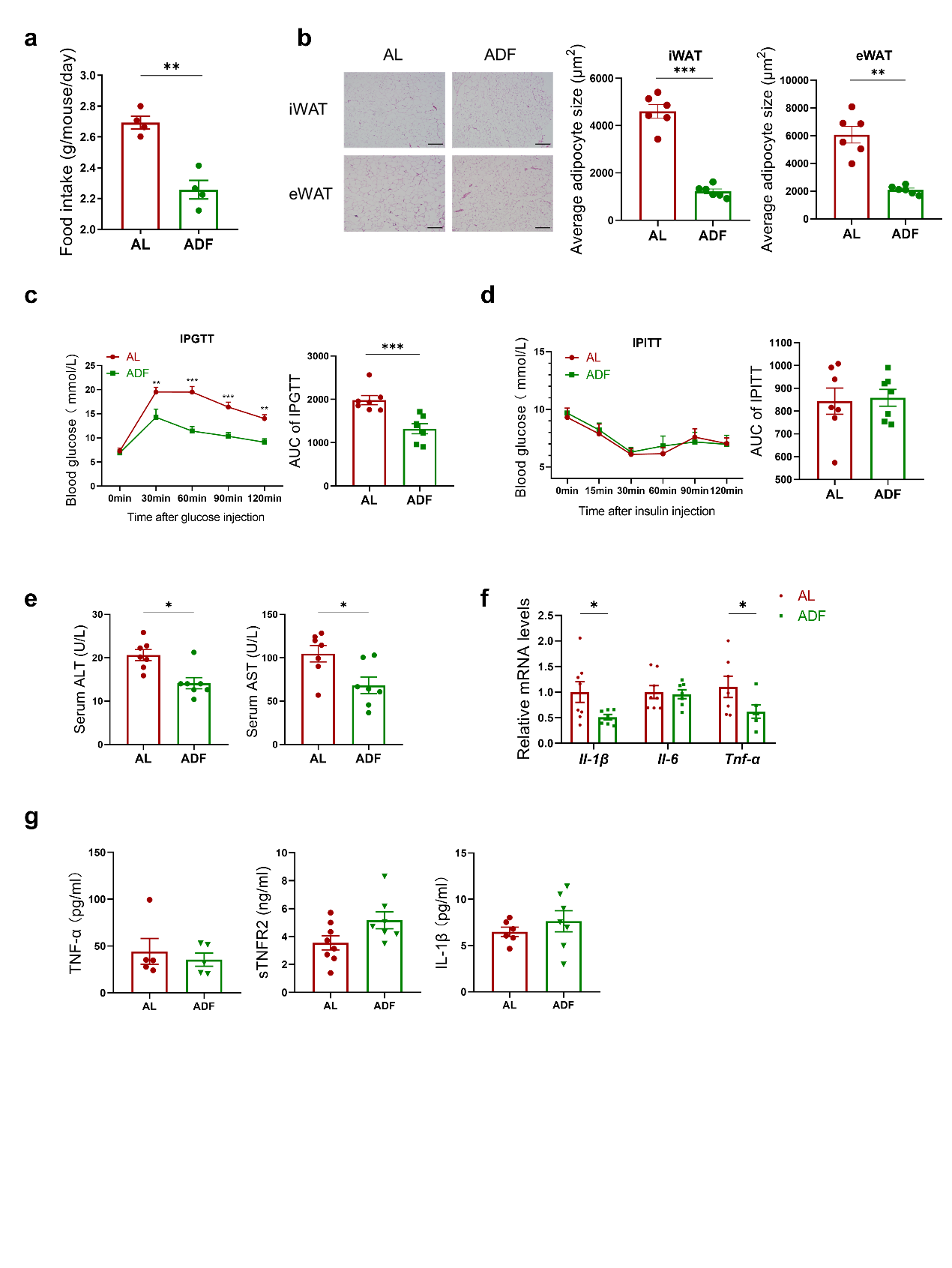


**Supplementary Figure S2** ADF ameliorates western diet-induced metabolic disorders of *Apoe^-/-^* mice. Eleven-week-old male *Apoe^-/-^* mice fed on WD were treated with either AL or ADF intervention for 16 weeks. (a) Average daily food intake (n=4 cages per group). (b) Representative H&E staining of epididymal white adipose tissue (eWAT) and subcutaneous inguinal white adipose tissue (iWAT) sections and quantifications of adipocyte size (n=6 mice per group). Scale bar(black), 100μm. (c) Intraperitoneal glucose tolerance test (ipGTT) (n=6 to 8 mice per group). (d) Intraperitoneal insulin tolerance test (ipITT) (n=6 to 8 mice per group). (e)Serum ALT and AST levels (n=6 to 8 mice per group). (f) Relative mRNA levels of inflammatory genes in the livers (n=6 to 8 mice per group). (g) The circulating levels of TNF-α, sTNFR2 and IL-1β were measured by ELISA (n=5 to 8 mice per group). Data are presented as mean±SEM. *P* values are determined by 2-tailed unpaired Student's *t*-test. **P* < 0.05, ***P* < 0.01, *** *P*< 0.001.


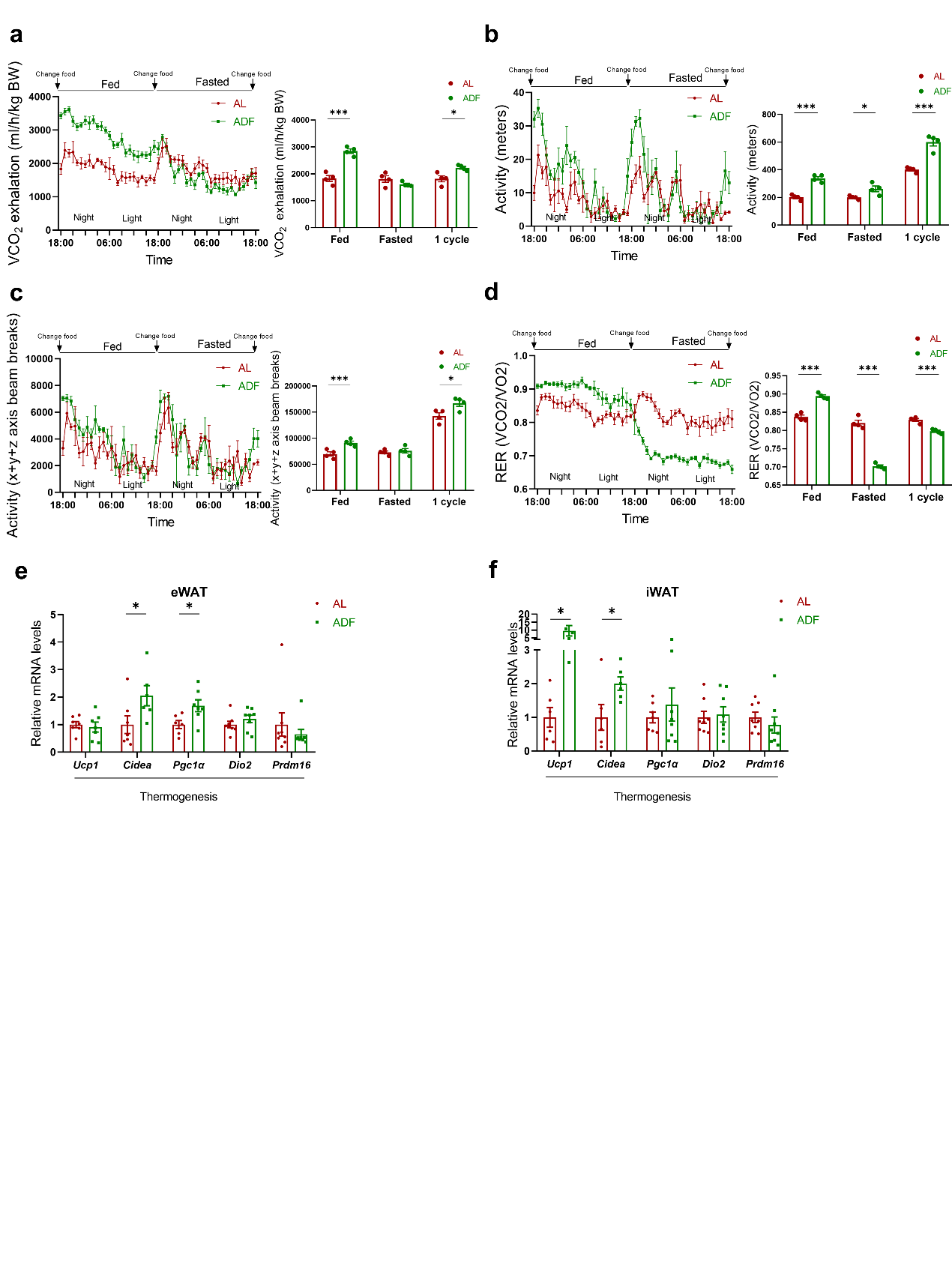


**Supplementary Figure S3** ADF promotes energy expenditure in *Apoe*^-/-^ mice. Eleven-week-old male *Apoe^-/-^* mice fed on WD were treated with either AL or ADF intervention for 16 weeks. (a-d) CO2 exhalation(a), locomotor activity (b and c) and respiratory exchange ratio (d) during one cycle of ADF (n=4 mice per group). CO2 exhalation was expressed relative to unit body weight. (e and f) The expressions of genes involved in thermogenesis in the eWAT (e) and iWAT(f) of AL and ADF mice evaluated by quantitative polymerase chain reaction (qPCR) (n=6 to 8 mice per group). Data are presented as mean±SEM. *P* values are determined by 2-tailed unpaired Student's *t*-test. **P* < 0.05, ***P* < 0.01, *** *P*< 0.001.


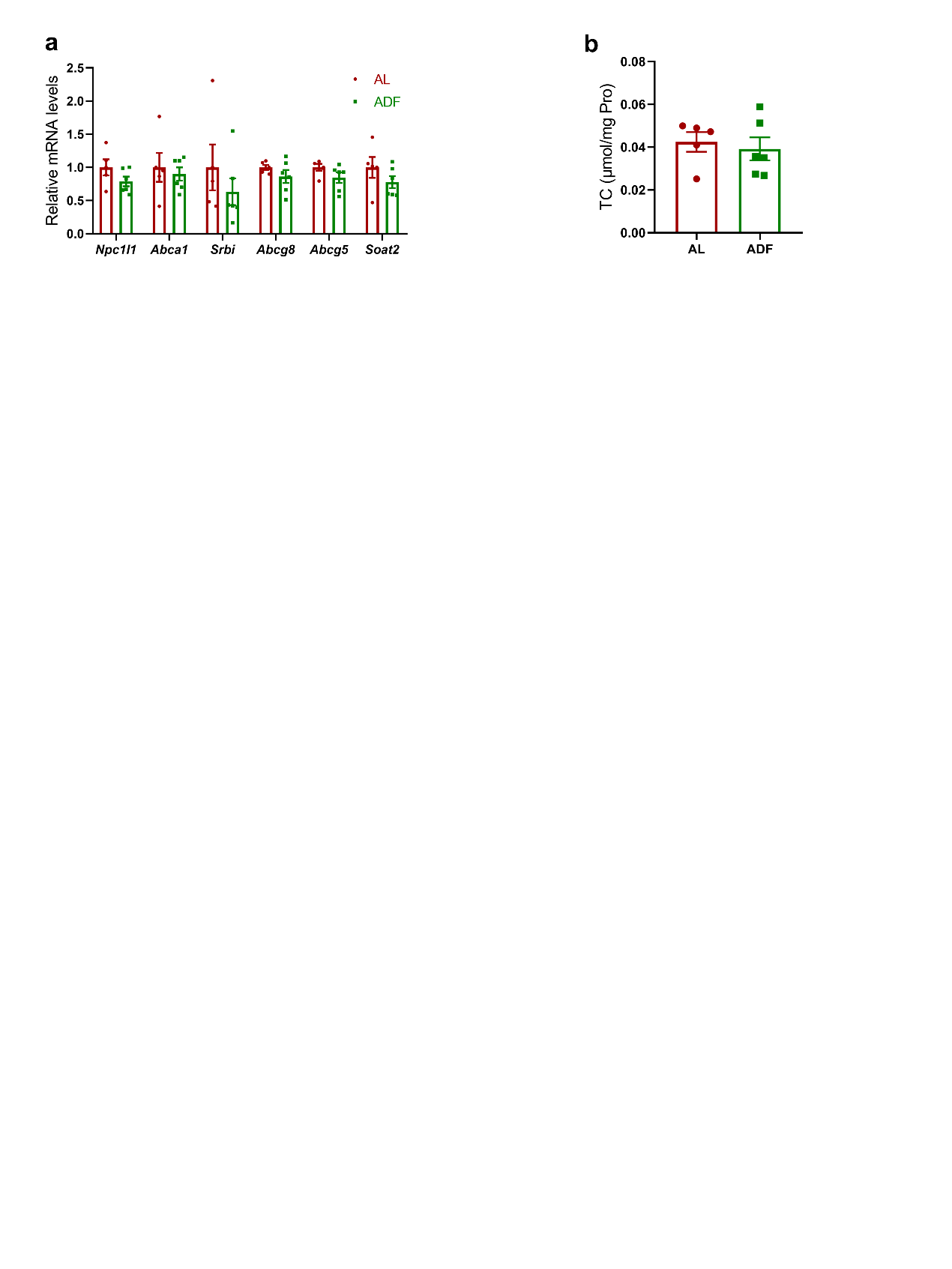


**Supplementary Figure S4** ADF has no effects on intestinal cholesterol absorption in *Apoe*^-/-^ mice. Eleven-week-old male *Apoe^-/-^* mice fed on WD were treated with either AL or ADF intervention for 16 weeks. (a and b) Relative mRNA levels of representative genes involved in cholesterol absorption(a) and total cholesterol levels(b) in proximal jejunum from AL and ADF mice (n=5 to 6 mice per group). Data are presented as mean ± SEM. *P* values are determined by 2-tailed unpaired Student's *t*-test.


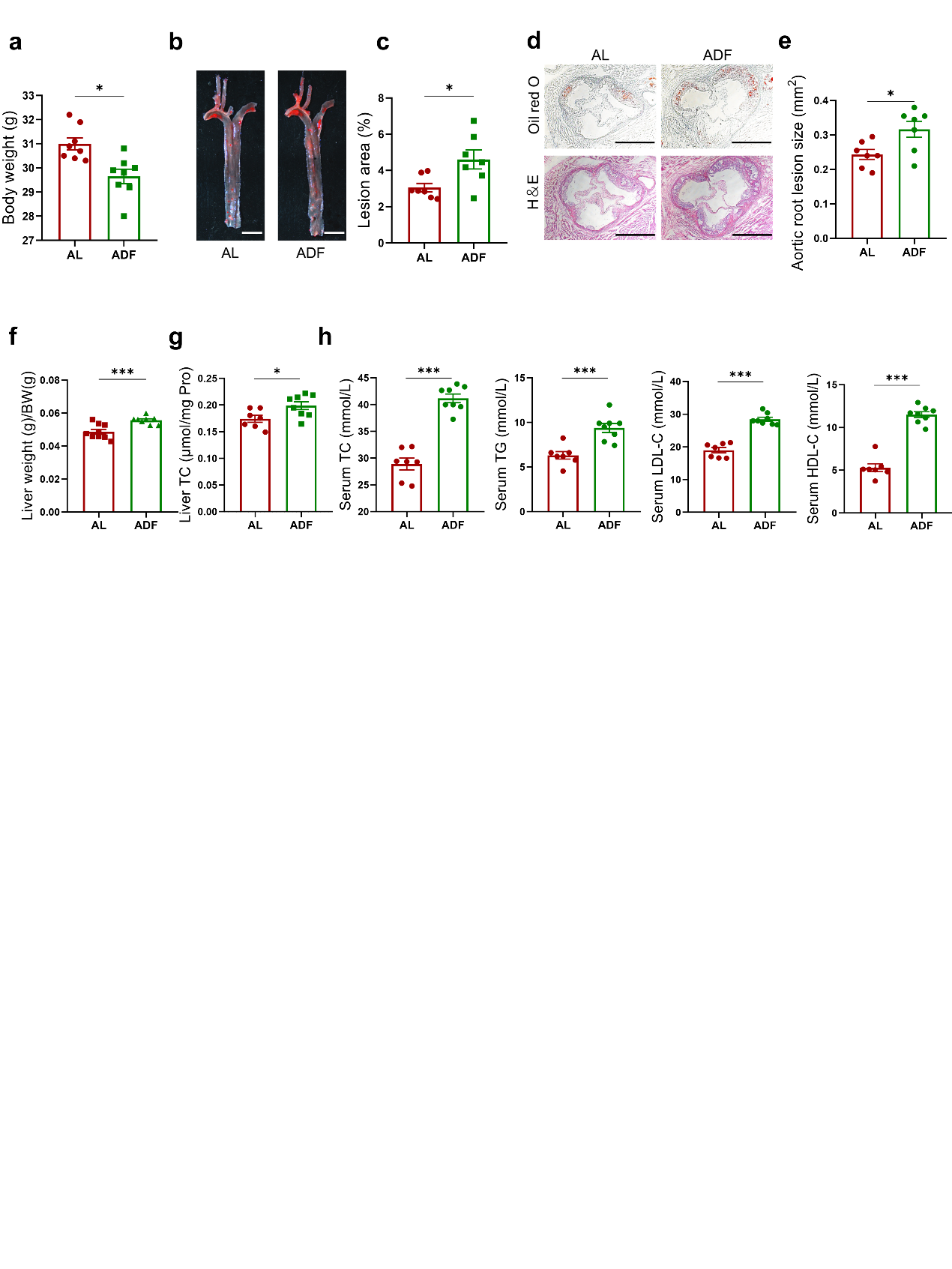


**Supplementary Figure S5** ADF aggravates cholesterol profile and atherosclerosis in female *Apoe*^-/-^ mice. Eleven-week-old female *Apoe^-/-^* mice fed on WD were treated with either AL or ADF intervention for 8 weeks. (a) Body weight measurement. (b) Representative microscopic photographs of en face Oil Red O staining of aortas in female *Apoe^-/-^* mice. Scale bar(white), 500μm. (c) Quantification of the en face atherosclerotic lesion areas of (b). (d) Representative histological analysis of Oil Red O and H&E staining of aortic root sections in female *Apoe^-/-^* mice fed with WD for 8 weeks. Scale bar(black), 500μm. (e) Quantification of the atherosclerotic lesion size of (d). (f) Liver weight-to-body weight ratio. (g)Total cholesterol contents in the liver. (h) Serum total cholesterol (TC), triglyceride (TG), low-density lipoprotein cholesterol (LDL-C) and high-density lipoprotein cholesterol (HDL-C) levels. Data are presented as mean ± SEM. n=7 to 8 mice per group. *P* values are determined by 2-tailed unpaired Student's *t*-test. **P* < 0.05, ***P* < 0.01, *** *P*< 0.001.


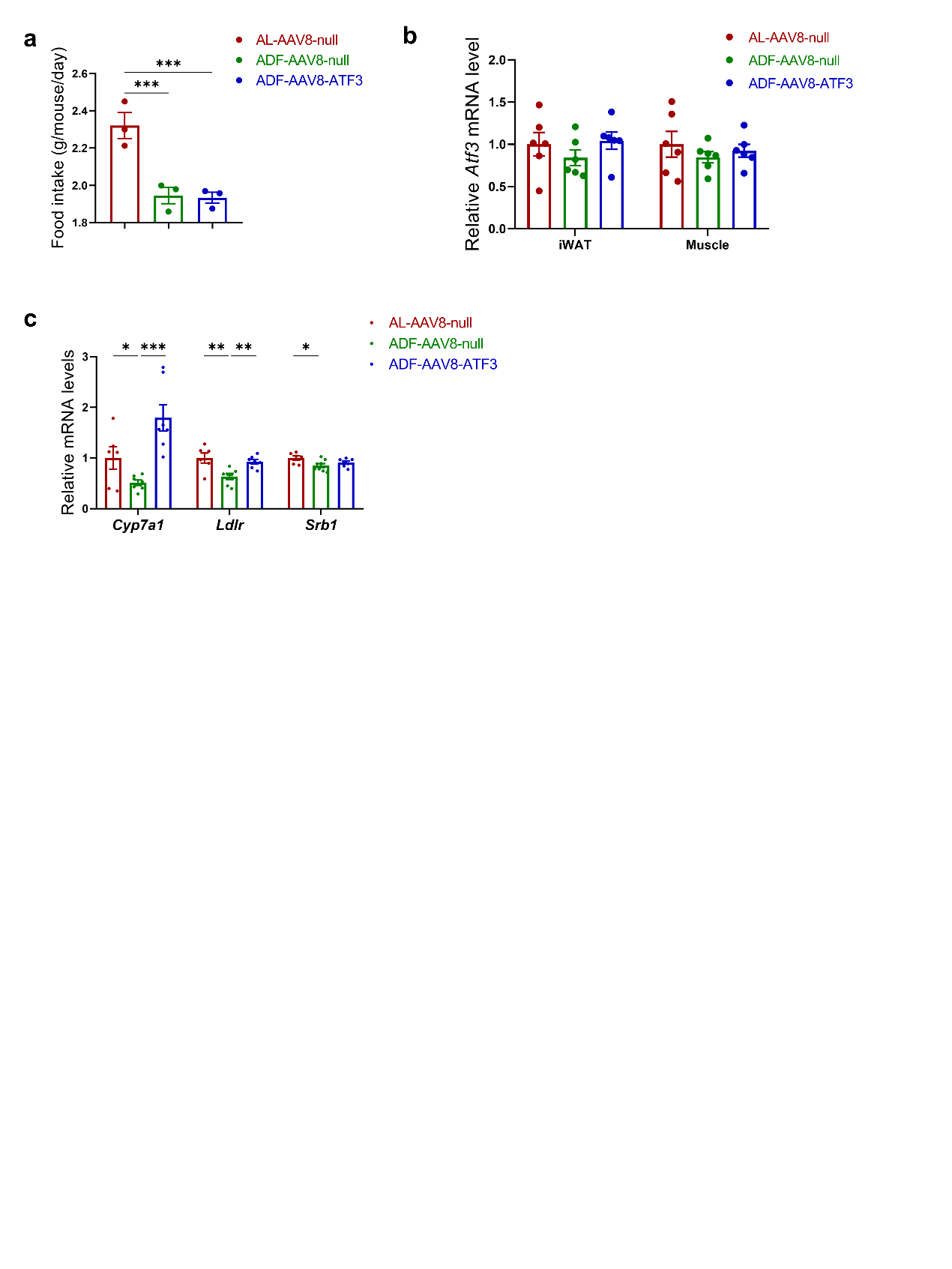


**Supplementary Figure S6** AAV8-ATF3 injection in ADF *Apoe*^-/-^ mice restores hepatic *Cyp7a1* and *Ldlr* expressions. Eleven-week-old male *Apoe^-/-^* mice were intravenously injected with either AAV8-TBG-null or AAV8-TBG-mATF3, and were then fed WD with AL or ADF regimen 1 week after injection. After 8 weeks of ADF regimen, mice were sacrificed. (a) Average daily food intake (n=3 cages per group). (b) Relative *Atf3* mRNA levels in iWAT and gastrocnemius muscle determined by qPCR (n =6 to 8 mice per group). (c) Relative mRNA levels of ATF3 target genes (n =6 to 8 mice per group). Data are presented as mean ± SEM. *P* values are determined by one-way ANOVA. **P* < 0.05, ***P* < 0.01, *** *P*< 0.001.

**Supplementary Tables**

**Supplementary Table S1** Primers designed for RT-qPCR

| Name | species | Sequence |
| --- | --- | --- |
| *36b4* | Mouse | F TGTTTGACAACGGCAGCATTT  R ACGCGCTTGTACCCATTGAT |
| *18S* | Mouse | F CGCCATGTCTCTAGTGATCC  R GGTCGATGTCTGCTTTCCTC |
| *Srebf2* | Mouse | F ACAACACTGACCAGCACCCAT  R GACGCTCAAGACAATCACACCA |
| *Hmgcr* | Mouse | F TGTTCACCGGCAACAACAAGA  R CACCGCGTTATCGTCAGGAT |
| *Hmgcs1* | Mouse | F AATGCCAGACCTACAGGTGGA  R CATGCTGCATGTGTGTCCCA |
| *Sm* | Mouse | F AGTTCGCTGCCTTCTCGGATA  R GCTCCTGTTAATGTCGTTTCTGA |
| *Fdft1* | Mouse | F GTTTGAAGACCCCATAGTTGGTG  R CACATCTACGTTCTCTGGCTTAG |
| *Lss* | Mouse | F CTCCAGAATGAGTTGGGTCGG  R CGCTTTTGGTAAGTCCGTGAAA |
| *Dhcr24* | Mouse | F ACCACTTCGTGGAAGGGTTG  R ACTGCCAATGCTATTCAGCTTG |
|  |  |  |
| *Fdps* | Mouse | F GGAGGTCCTAGAGTACAATGCC  R AAGCCTGGAGCAGTTCTACAC |
| *Pmvk* | Mouse | F GACTTCTGGATGCGAGCACCT  R CTGGGACACGCCTTCCACAATC |
| *Mvk* | Mouse | F CTGACCTCCATTGACGCAATATC  R CCACCCCGAGAGCATTCAG |
| *Mvd* | Mouse | F ATGGCCTCAGAAAAGCCTCAG  R TGGTCGTTTTTAGCTGGTCCT |
| *Nsdhl* | Mouse | F TGGAGCGAGGCTATACTGTCA  R TGTTGTTACTGTACGGCGGAGG |
| *Abca1* | Mouse | F GCTTGTTGGCCTCAGTTAAGG  R GTAGCTCAGGCGTACAGAGAT |
| *Abcg1* | Mouse | F CTTTCCTACTCTGTACCCGAGG  R CGGGGCATTCCATTGATAAGG |
| *Abcg5* | Mouse | F CCAGATTATGTGCATCTTAGGCA  R CTGCTCAGAAAAACGTCGCT |
| *Abcg8* | Mouse | F CTCCTTCTTCTGCAATGCCCTC  R ATCCATGCAGGCACTATCCAC |
| *Soat2* | Mouse | F ACAAGACAGACCTCTTCCCTC  R ATGGTTCGGAAATGTTGCACC |
| *Ldlr* | Mouse | F TCAGACGAACAAGGCTGTCC  R CCATCTAGGCAATCTCGGTCTC |
| *Atf3* | Mouse | F ATAAACACCTCTGCCATCGG  R GCCTCCTTTTCCTCTCATCTTC |
| *Klf6* | Mouse | F GCACGAAACGGGCTACTTCTC  R AGAGTCCTCTGGGGGACTAGA |
| *Atf4* | Mouse | F AAGGAGGAAGACACTCCCTCT  R CAGGTGGGTCATAAGGTTTGG |
| *Sort1* | Mouse | F TGGGGTTATTCTCGTCCTGAC  R CCGTCCGAATGAAGGTGTTATTG |
| *Cyp7a1* | Mouse | F AGCAACTAAACAACCTGCCAGTA  R TGTCCGGATATTCAAGGATGCAC |
| *Scarb1* | Mouse | F TGTACTGCCTAACATCTTGGTCC  R CACAGGATCTCACCAACTGTGC |
| *Cyp8b1* | Mouse | F CTAGGGCCTAAAGGTTCGAGT  R GTAGCCGAATAAGCTCAGGAAG |
| *Foxq1* | Mouse | F ACTGATGACAGCAGAACGCA  R AGGTGTATTCGCTGTTGGGG |
| *D130040H23Rik* | Mouse | F ACAACAGAGTCACCTCCAGAT  R GTGATGTGCAAAGGTCTTACCA |
| *Bhlha15* | Mouse | F GCTGACCGCCACCATACTTAC  R TGTGTAGAGTAGCGTTGCAGG |
| *Nr4a3* | Mouse | F AGGATTCACTGATCTCCCCAA  R GATGCAGGACAAGTCCATTGC |
| *Id1* | Mouse | F GGTCCGAGGCAGAGTATTACA  R CCTGAAAAGTAAGGAAGGGGGA |
| *Grhl1* | Mouse | F CCGTGCTGGTACTTCAGAATG  R GCTGTCTTCGTCTCCGTTG |
| *Srebf1* | Mouse | F GCAGCCACCATCTAGCCTG  R CAGCAGTGAGTCTGCCTTGAT |
| *Prrx1* | Mouse | F GAGCGTGTCTTTGAGCGGA  R CATGTGGCAGAATAAGTAGCCAT |
| *Zfp979* | Mouse | F AGACATAAAACTCGGACCA  R TTTCTTCTCTATGTGGGTT |
| *Spic* | Mouse | F AAACATTTCAAGACGCCATTGAC  R CTCTGACGTGAGGATAAGGGT |
| *Trps1* | Mouse | F GGTACAGAGGCCACCAGTTAT  R GGCTCTCCTTCTACACTTTTGG |
| *Elf4* | Mouse | F AACGTGTCATCCACTGAAGTC  R TCAGGGGTAGAGAGCAGGAAG |
| *Mlxipl* | Mouse | F AGATGGAGAACCGACGTATCA  R ACTGAGCGTGCTGACAAGTC |
| *Tox* | Mouse | F GCTCCCGTTCCATCCACAAA  R TCCCAATCTCTTGCATCACAGA |
| *Cidea* | Mouse | F GGTGGACACAGAGGAGTTCTTTC  R CGAAGGTGACTCTGGCTATTCC |
| *Ucp1* | Mouse | F AGGCTTCCAGTACCATTAGGT  R CTGAGTGAGGCAAAGCTGATTT |
| *Pgc-1α* | Mouse | F AAGTGGTGTAGCGACCAATCG  R AATGAGGGCAATCCGTCTTCA |
| *Dio2* | Mouse | F GGTGGTCAACTTGGTTCAGCC  R AAGTCAGCCACCGAGGAGAACT |
| *Prdm16* | Mouse | F ATCCACAGCACGGTGAAGCCAT  R ACATCTGCCACAGTCCTTGCA |
| *Tnf-α* | Mouse | F AGTCCGGGCAGGTCTACTTT  R TTGGACCCTGAGCCATAATC |
| *Il-1β* | Mouse | F GAAATGCCACCTTTTGACAGTG  R TGGATGCTCTCATCAGGACAG |
| *Il-6* | Mouse | F TAGTCCTTCCTACCCCAATTTCC  R TTGGTCCTTAGCCACTCCTTC |
| *Npc1l1* | Mouse | F CGCCCTTCTTTCTACATGGGT  R GAATCTGCGCTTACGAGGGAG |

**Supplementary Table S2** Antibodies list

| Name | Supplier | Cat no. | Dilution | Clone no. |
| --- | --- | --- | --- | --- |
| PERK | ABclonal | A18196 | 1:1000 | Polyclonal |
| P-PERK | ABclonal | AP0886 | 1:1000 | Polyclonal |
| eIF2α | ABclonal | A9709 | 1:1000 | ARC1712 |
| P-eIF2α | Proteintech | 28740-1-AP | 1:1000 | Polyclonal |
| ATF3 | ABclonal | A13469 | 1:1000 | Polyclonal |
| ATF4 | ABclonal | A21500 | 1:1000 | Polyclonal |
| KLF6 | Proteintech | 14716-1-AP | 1:1000 | Polyclonal |
| HSP90 | Proteintech | 13171-1-AP | 1:5000 | Polyclonal |
